# E-Learning Modules Based on Bloom Taxonomy and the Miller Pyramid for First-Year Indian Medical Students: Randomized Controlled Study in Medical Education

**DOI:** 10.2196/84339

**Published:** 2026-04-07

**Authors:** Archana Prabu Kumar, Padmavathi R, Maheshkumar K, Abirami Omprakash, Prabu Kumar Chokkalingam Mani, B W C Sathiyasekaran, Maruthy K N

**Affiliations:** 1 Medical Education Department, College of Medicine and Health Sciences, Arabian Gulf University, Bahrain, Bahrain; 2 Department of Physiology, Sri Ramachandra Medical College and Research Institute Sri Ramachandra Institute of Higher Education and Research, No 1 Ramachandra Nagar, Chennai, Tamil Nadu, 600116, India, 91 09710404913; 3 Department of Physiology and Biochemistry, Government Yoga and Naturopathy Medical College and Hospital, Chennai, Tamil Nadu, India; 4 Department of Pathology, Government Hospitals – Salmaniya Medical Complex, Manama, Bahrain; 5 Sree Balaji Medical College, Chennai, Tamil Nadu, India; 6 Department of Physiology, PES Institute of Medical Sciences and Research, Kuppam, Tamil Nadu, India

**Keywords:** competency-based medical education, CBME, e-module, Bloom taxonomy, Miller pyramid, medical undergraduates, digital learning

## Abstract

**Background:**

Competency-based medical education (CBME) in India emphasizes early competency formation, higher-order cognitive processing, and self-directed learning. Although e-learning is widely adopted, there is limited evidence on structured e-modules explicitly designed using Bloom taxonomy and the Miller pyramid for Indian MBBS students.

**Objective:**

This study aims to design, validate, implement, and evaluate CBME-aligned e-modules for first-year MBBS foundational subjects (anatomy, physiology, and biochemistry) and to compare their effectiveness with traditional teaching on cognitive, psychomotor, and affective learning outcomes using Kirkpatrick levels 1 and 2.

**Methods:**

A randomized controlled study was conducted among 690 first-year medical undergraduates (control: n=370; intervention: n=320). The intervention group received Sharable Content Object Reference Model–based interactive e-modules through Moodle (Modular Object-Oriented Dynamic Learning Environment) in addition to standard lectures, while the control group received lectures only. e-Modules were designed using Bloom taxonomy and the Miller pyramid, validated by internal and external experts, and implemented following Kern 6-step approach. Learning outcomes were assessed using structured feedback (Kirkpatrick level 1) and end-of-block internal assessments comprising multiple choice questions, short notes, and objective structured clinical examinations (Kirkpatrick level 2).

**Results:**

Students in the intervention group performed significantly better across all cognitive levels compared with the control group: remember (mean 6.52, SD 2.24 vs mean 5.26, SD 2.89; *P*<.001), understand (mean 2.92, SD 1.45 vs mean 2.55, SD 1.13; *P*<.001), apply (mean 3.43, SD 1.06 vs mean 2.56, SD 1.08; *P*<.001), and analyze (mean 3.01, SD 0.83 vs mean 2.53, SD 1.11; *P*<.001). Psychomotor scores (objective structured clinical examination manipulation: mean 4.55, SD 1.12 vs mean 4.10, SD 1.42; *P*<.001) and affective domain scores (mean 3.02, SD 0.81 vs mean 2.03, SD 0.81; *P*<.001) were also significantly higher in the intervention group. Subgroup analysis showed the largest gains among medium achievers across domains.

**Conclusions:**

CBME-aligned e-modules significantly enhanced student performance in cognitive, psychomotor, and affective domains compared with traditional teaching alone, with particularly pronounced benefits for medium achievers. Well-designed e-modules represent a scalable, adaptable strategy to support CBME implementation across diverse medical education settings in India.

## Introduction

### Background

The National Medical Commission in India introduced a new approach, known as competency-based medical education (CBME), in 2015 and officially implemented it in 2019 [1]. This updated curriculum encourages new ways of learning, including self-directed learning; elective modules; and training in attitude, ethics, and communication [2,3]. It is aimed at directing medical education to focus more on helping students build essential skills, such as critical thinking and decision-making.

While traditional lectures often struggle to maintain student interest and foster deeper understanding and critical reasoning skills [4,5], e-learning (computer-assisted learning), which uses electronic devices to support teaching and learning, is becoming increasingly popular [6]. A common way e-learning helps is through blended learning, which mixes classroom teaching with online materials, such as tutorials [7]. One such tool is e-modules. An e-module is defined as an e-learning strategy, which focuses on 1 or 2 learning concepts, usually incorporating a blend of teaching and assessment tools that may include multimedia, video clips, direct lecture instruction, game-based activities, and even links to social media [8,9] to help students stay engaged and learn better [7]. Such tools have been shown to improve short-term memory and keep learners engaged [10]. They are found useful in early medical training, where complex theory can be better understood through interactive content [11]. However, for e-learning to be successful, it must consider cognitive limitations and learning preferences [12].

### Rationale

The COVID-19 pandemic led to a rapid increase in digital learning tools [13], and research over time has shown that these tools can help students gain both knowledge and skills [14,15]. CBME is based on well-known learning frameworks, such as Bloom taxonomy [16] and the Miller pyramid [17], which help structure learning in a step-by-step way. Yet, the use of digital modules built on these frameworks is limited [18]. There is a clear gap, and few studies have developed or tested e-modules created specifically for Indian medical students, especially those based on Bloom and Miller models [19].

Moreover, to evaluate training programs, studies in the past have not applied comprehensive evaluations of the Kirkpatrick model to assess e-learning interventions in medical education [20]. A study evaluating a teaching workshop for health care staff has used the first 3 levels, demonstrating improvements in satisfaction, knowledge, and behavior [8]. Such evaluations specifically targeting e-learning modules for Indian medical undergraduates are limited.

### Study Objectives

Thus, this study aimed to develop, validate, implement, and evaluate e-modules tailored for Indian medical undergraduates. The objectives of the study are as follows:

1. To design e-modules for first-year MBBS foundational disciplines (anatomy, physiology, and biochemistry), using Bloom taxonomy and the Miller pyramid to target cognitive, affective, and psychomotor competencies outlined in the Indian CBME curriculum.
2. To evaluate the effectiveness of these e-modules, implemented through Moodle-SCORM (Modular Object-Oriented Dynamic Learning Environment–Sharable Content Object Reference Model), in comparison with traditional classroom teaching by examining learning outcomes across cognitive, psychomotor, and affective domains using Kirkpatrick levels 1 and 2.

This study provides one of the first large-scale evaluations of theory-driven e-modules designed specifically for Indian medical undergraduates, addressing a critical gap in CBME-aligned digital learning research. By addressing these objectives, this study seeks to bridge the gap between pedagogical strategies and technological advancements in medical education, ultimately enhancing the competency and preparedness of future health care professionals.

## Methods

### Study Design and Setting

This randomized controlled study was conducted between 2015 and 2018 at a tertiary medical university in south India. The study followed the Kern 6-step approach [7,21] for curriculum development and aimed to evaluate the effectiveness of e-learning modules, designed using Bloom taxonomy and the Miller pyramid, in enhancing learning outcomes among first-year MBBS students.

### Ethical Considerations

This study received ethical approval from the Institutional Ethics Committee of Sri Ramachandra University, Chennai, India (Approval No: IEC-NI/12/OCT/30/53, approved on March 24, 2014). All procedures were conducted in accordance with the ethical standards of the institutional review board and the principles of the Declaration of Helsinki.

Participation in the study was voluntary, and all participants were informed about the purpose and procedures of the study prior to participation. Written informed consent was obtained from all participants. Participant privacy and confidentiality were strictly maintained. Data were anonymized prior to analysis, and no personally identifiable information was included in the dataset used for analysis or in the reporting of results. Participants did not receive any financial compensation or incentives for their participation in this study.

### Participants and Randomization

A total of 690 first-year MBBS students enrolled during the study period were invited to participate. Students who completed all baseline assessments and attended the scheduled end-of-block internal assessments (EBIAs) were included in the final analysis.

Students were randomized into control and intervention groups using the Research Randomizer software (version 4.0; Geoffrey C. Urbaniak and Scott Plous; Multimedia Appendix 1).

- Control group: This group received standard PowerPoint (Microsoft Corp) lectures
- Intervention group: This group received both PowerPoint lectures and e-modules via Moodle with unique login credentials (Multimedia Appendix 2)

All students in the control group gained access to e-modules after the end-of-block assessment to ensure equitable learning opportunities.

**Figure 1.**
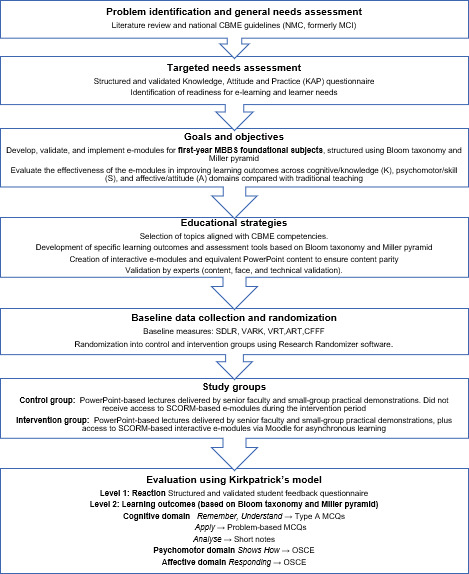
Flowchart of curriculum development, implementation, and evaluation steps based on Kern 6-step approach and the Kirkpatrick model. ART: auditory reaction time; CBME: competency-based medical education; CFFF: critical flicker fusion frequency; MCI: Medical Council of India; NMC: National Medical Commission; OSCE: objective structured clinical examination; SDLR: self-directed learning readiness; SLO: specific learning outcomes; VARK: visual auditory reading kinesthetic; VRT: visual reaction time.

The steps followed for data collection are shown in Figure 1.

### Targeted Needs Assessment

The data were collected using a structured and validated knowledge, attitude, and practice questionnaire (Multimedia Appendix 3) [6,10]. Knowledge, attitude, and practice analysis revealed high digital readiness, with all students owning internet-enabled devices, most confident in basic software use and strongly favoring interactive e-modules in education.

The results emphasized the need to develop self-directed e-learning tools tailored to the diverse learning styles of Indian medical undergraduates. E-learning strategies were identified as essential for the rapid and effective implementation of new curricula.

### Educational Intervention

#### Selection of Topics

Four topics were selected based on global or national significance, core learning relevance, feasibility, faculty expertise, and alignment with the curriculum. A list of e-modules addressing the specific CBME competencies and learning objectives aligned with respective assessment tools was categorized under Bloom taxonomy and the Miller pyramid (Multimedia Appendix 4). The selected e-modules covered core foundational topics from anatomy, physiology, and biochemistry that map directly to first-year CBME competencies.

#### Development of e-Modules

e-Modules were developed as structured, interactive learning tools aligned with CBME competencies. Content (text, images, and animations) was created collaboratively by subject experts and educational technologists using standard multimedia authoring software. Visual elements were sourced from textbooks and open-access repositories in accordance with fair-use guidelines, and additional illustrations were created where needed (Multimedia Appendix 5).

#### Platform (Moodle-SCORM) and Delivery Approach

Multimedia components, including narration, animations, and interactive elements, were combined to produce SCORM-compliant packages suitable for delivery via Moodle. Each module included embedded self-assessment exercises (multiple choice questions [MCQs], case scenarios, and concept-based questions) with automated scoring and immediate feedback (MultimediaAppendices 6  7).

To ensure content equivalence, identical learning material (text and images) was used to create accompanying PowerPoint presentations for the control group, thereby avoiding content bias between instructional formats.

#### Validation of Learning Materials

The e-modules and PowerPoint were proofread and face-validated by students (second-year undergraduate students who were not part of this study). Content validation and technical validation were done by 2 internal and 2 external experts, following a structured checklist (Multimedia Appendix 8). Suggestions by the students and experts were incorporated wherever possible and feasible. There were certain similarities and differences between PowerPoint (2016) and the e-modules used in this study. Both platforms made use of text, images, audio, video, and animations. However, e-modules offered several interactive features over PowerPoint, such as interactive images, easy navigation with a toolbar or index, self-assessment with specific feedback, competitive scoring, and access restrictions based on performance. Additionally, e-modules enabled the tracking of student performance and the generation of reports, making them a more dynamic and data-driven learning tool compared to PowerPoint. Modules were converted into Sharable Content Object Reference Model (SCORM, 2016) format for integration into a Moodle (2016) [22].

#### Copyright and Intellectual Property

The developed e-modules were submitted for copyright registration and granted under L-83129/2019.

### Baseline Measures

Before implementation, the data on self-directed learning readiness [23] and learning styles (Visual, Aural, Read/Write, and Kinesthetic questionnaire version 7.8) [24], visual reaction time, auditory reaction time, and critical flicker fusion frequency were collected. These measures were used to describe the learner profile and explore potential associations with learning outcomes, without influencing group allocation or intervention intensity. Prior authorization was obtained for administering these questionnaires (Multimedia Appendix 9). Although the self-directed learning readiness scale was administered during the study period (2015‐2018), we have cited the 2021 validation study as the most current reference establishing construct validity in Indian MBBS learners. The scale itself predates this reference, and no post-2018 findings were incorporated into data collection or analysis.

### Assessment Framework

The effectiveness of e-modules was based on the Kirkpatrick learning evaluation model [25]. Table 1 shows the different levels of the Kirkpatrick learning evaluation model, learning domains, and corresponding tools of evaluation used in this study.

**Table 1.** Evaluation of the effectiveness of e-modules based on the Kirkpatrick learning evaluation model.

Kirkpatrick model and domain		Taxonomy Bloom level	Taxonomy Miller pyramid	Tools for evaluation
Level 1: Reaction				
	—^a^	—	—	Structured and validated feedback questionnaire containing open- and close-ended questions
Level 2: Learning				
	Cognitive	Remember Understand Apply Analyze	Knows Knows how Knows how Knows how	MCQs^b^^,^^c^ Problem-based MCQs^b^ Problem-based MCQs^b^ Short notes^b^
Attitude	Responding	Shows how	OSCE^d^
Skill	Manipulation^e^	Shows how	OSCE

a Not applicable.

b End of block internal assessment.

c MCQs: multiple choice questions.

d OSCE: objective structured clinical examination.

e Classification by Dave, 1970 [26].

#### Level 1: Evaluation of Reaction

The students’ reactions were evaluated using a structured and validated feedback questionnaire containing open- and closed-ended questions (Multimedia Appendix 10). The quantitative results of the evaluation of reaction and qualitative data were transcribed word by word verbatim in a Word document and analyzed thematically using a deductive coding approach (Multimedia Appendix 11).

#### Level 2: Evaluation of Learning

Evaluation of learning was assessed through MCQs (designed appropriately to assess “Remember,” “Understand,” and “Apply” levels of Bloom taxonomy) and short notes (designed to assess “Analysis” level) in the EBIA.

#### Standardization of EBIA

Fifteen MCQs (combination of levels of remember, understand, and application), 4 short notes (analysis level), and 1 structured essay totaled 50 marks. Question papers followed standardized blueprints and were administered within 90 minutes under invigilated conditions. The difficulty index was comparable with other EBIAs held during the academic year.

### Outcome Measures and Scoring Procedures

Each internal assessment was graded by a single designated faculty member, as per the examination policy of the host university. All faculty involved in this research were exempted from any duty related to assessment and evaluation as per the examination or research ethics policy of the host university. This practice was intentionally adopted to maintain scoring consistency and minimize variability or rater-related bias (eg, halo effect). To further ensure fairness and uniformity, all answer sheets were anonymized using dummy roll numbers prior to evaluation. Additionally, the evaluating faculty member was provided with a standardized marking guide (answer key or scoring rubric) that detailed the expected key points and the corresponding marks to be awarded. This ensured transparent and objective grading across all responses.

### Statistical Analysis

The data were analyzed using standard descriptive and inferential statistics. Continuous variables were summarized as means and SDs. Group comparisons were performed using independent 2-tailed *t* tests.

Effect sizes (Cohen *d*) were calculated to quantify the magnitude of differences between the groups across cognitive, psychomotor, and affective domains. Subgroup analyses were conducted to examine differential effects among high, medium, and low academic achievers. Students were categorized into low achievers (<40% in previous assessments) and high achievers (>70% in previous assessments) to analyze differential learning impacts within the control and intervention groups. A significance level of *P*<.05 was adopted for all analyses.

## Results

### Participant Characteristics

**Figure 2.**
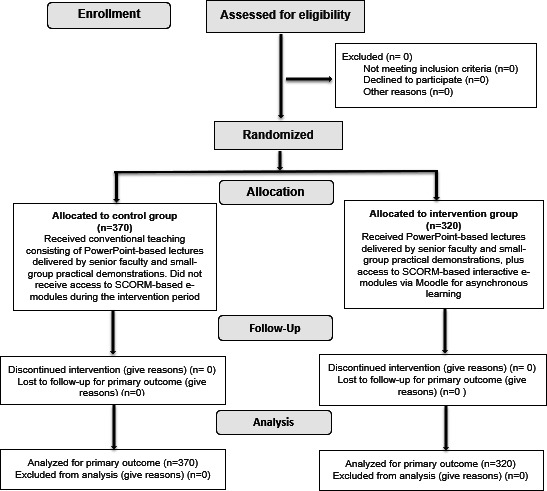
CONSORT (Consolidated Standards of Reporting Trials) 2025 flow diagram. Flow diagram of the progress through the phases of a randomized trial of 2 groups (ie, enrollment, intervention allocation, follow-up, and data analysis). Moodle: Modular Object-Oriented Dynamic Learning Environment; SCORM: Sharable Content Object Reference Model [27].

A total of 690 first-year undergraduate students participated in this study. The steps followed in participant enrollment, allocation, follow-up, and analysis are shown in Figure 2. The participants randomly assigned to the control groups were comparable in terms of baseline characteristics (Table 2). All students completed baseline assessments and the EBIAs.

**Table 2.** Demographic details of study participants.

Characteristic	Overall (N=690)	Control (n=370)	Intervention (n=320)	*P* value
Gender, n (%)				*.*06
Male	272 (39.4)	149 (40.3)	123 (38.4)	
Female	418 (60.6)	221 (59.7)	197 (61.6)	
Attendance, mean (SD)	84.70 (8.06)	84.82 (7.93)	84.55 (8.22)	*.*03
Academic performance, mean (SD)	54.94 (12.20)	54.36 (12.43)	55.61 (11.92)	*.*02
Level of achievers, n (%)				*.*06
High	89 (12.9)	44 (11.9)	45 (14.1)	
Low	114 (16.5)	65 (17.6)	49 (15.3)	
Medium	487 (70.6)	261 (70.5)	226 (70.6)	
Visual reaction time (ms), mean (SD)	—^a^	284 (92)	240 (71)	*.*07
Auditory reaction time (ms), mean (SD)	—	236 (105)	212 (61)	*.*07
Critical flicker fusion frequency (Hz), mean (SD)	—	27 (3)	28 (5)	*.*06
Self-directed learning readiness, mean (SD)	—	156 (12)	153 (14)	*.*09

a Not applicable.

### Kirkpatrick Level 1: Student Reactions

During level 2, the intervention group received e-modules in addition to traditional lectures and outperformed the control group across all cognitive domains. The intervention group showed superior performance in motor and affective domains, indicating that e-modules significantly improved student performance not only in knowledge acquisition but also in practical skills and attitudinal engagement. Table 3 shows that students in the intervention group consistently outperformed the control group across all cognitive levels. The largest differences were observed in the apply and analyze domains, indicating that the e-modules were particularly effective in promoting higher-order thinking. All comparisons were statistically significant (*P*<.001), demonstrating a meaningful advantage of the blended e-learning approach.

**Table 3.** Evaluation of the effectiveness of e-modules on Kirkpatrick model level 2: learning.

Domain^a^ and level		Total (N=690)	Control (n=370)	Intervention (n=320)	Effect size (Cohen *d*)	*P* value
Knowledge, mean (SD)						<.001
Remember (direct MCQs^b^, 10 marks)		5.84 (2.68)	5.26 (2.89)	6.52 (2.24)	0.48	
Understand (concept-based MCQs, 5 marks)		2.73 (1.30)	2.55 (1.13)	2.92 (1.45)	0.29	
Application (problem-based MCQs, 5 marks)		2.96 (1.15)	2.56 (1.08)	3.43 (1.06)	0.81	
Analysis (SAQ^c^, 5 marks)		2.75 (1.02)	2.53 (1.11)	3.01 (0.83)	0.48	
Motor, mean (SD)						
Manipulation (OSCE^d^, 6 marks)		4.31 (1.31)	4.10 (1.42)	4.55 (1.12)	0.35	<.001
Affective, mean (SD)						
Responding (OSCE, 4 marks)		2.49 (0.96)	2.03 (0.82)	3.02 (0.81)	1.21	<.001

a Comparison of scores across cognitive (Bloom’s 4 levels), motor (manipulation, Dave level), and affective (responding, Bloom domain) between the control and intervention groups.

b MCQ: multiple choice question.

c SAQ: short answer question.

d OSCE: objective structured clinical examination.

### Kirkpatrick Level 2: Learning Outcomes

#### Cognitive Outcomes

Students who received the e-modules demonstrated significantly higher performance across all cognitive levels assessed (Table 3). Effect sizes ranged from small to large: remember (*d*=0.48; 6.52 vs 5.26; *P*<.001), understand (*d*=0.29; 2.92 vs 2.55; *P*<.001), application (*d*=0.81; 3.43 vs 2.56; *P*<.001), and analysis (*d*=0.48; 3.01 vs 2.53; *P*<.001). The large effect size for application skills indicates that e-modules were particularly effective in enhancing students’ problem-solving abilities.

#### Psychomotor Outcomes

The intervention group demonstrated superior psychomotor performance (*d*=0.35; 4.55 vs 4.10; *P*<.001), suggesting that visual demonstrations and procedural guidance in e-modules effectively enhanced motor skill acquisition.

#### Affective Outcomes

Affective skills showed the most substantial improvement (*d*=1.21; 3.02 vs 2.03; *P*<.001), with the largest effect size across all domains. This highlights the exceptional value of structured e-modules with visual explanations, role-modeling, and repeated exposure to professional behaviors for developing interpersonal competencies and professional attitudes—outcomes traditionally challenging to achieve through conventional teaching.

### Subgroup Analysis

The impact of e-modules was found to vary across different achievement groups (Table 4). Subgroup analysis revealed that the intervention was particularly effective among medium achievers, showing significant improvements across all domains (*P*<.001 for all comparisons). High achievers in the intervention group showed significant improvements in application (*P*<.001) and affective domains (*P*<.001). Low achievers demonstrated significant improvements in understanding (*P*<.001), application (*P*<.001), analysis (*P*=.01), and affective domains (*P*<.001).

**Table 4.** Evaluation of the effectiveness of e-modules on Kirkpatrick model level 2: learning.

Domain^a^ and level		High achievers			Low achievers			Medium achievers		
		Control	Intervention	*P* value	Control	Intervention	*P* value	Control	Intervention	*P* value
Knowledge, mean (SD)										
Remember (Direct MCQ^b^s, 10 marks)		5.14 (2.72)	6.18 (2.10)	.04	5.49 (2.87)	5.88 (1.96)	.39	5.22 (2.93)	6.73 (2.29)	<.001
Understand (Concept-Based MCQs, 5 marks)		2.45 (1.11)	2.58 (1.25)	.62	2.49 (1.16)	3.65 (1.60)	<.001	2.59 (1.13)	2.84 (1.41)	=.03
Application (Problem-Based MCQs, 5 marks)		2.55 (1.04)	4.38 (0.49)	<.001	2.63 (1.13)	3.63 (0.49)	<.001	2.54 (1.07)	3.20 (1.12)	<.001
Analysis (SAQ^c^, 5 marks)		2.16 (1.12)	2.80 (0.84)	.003	2.62 (1.11)	3.08 (0.79)	.01	2.57 (1.11)	3.04 (0.83)	<.001
Motor, mean (SD)										
Manipulation (OSCE^d^, 6 marks)		4.02 (1.47)	4.56 (1.03)	.05	4.00 (1.46)	4.47 (1.10)	.05	4.14 (1.40)	4.57 (1.15)	<.001
Affective, mean (SD)										
Responding (OSCE, 4 marks)		2.48 (0.76)	3.16 (0.74)	<.001	1.77 (0.77)	3.08 (0.76)	<.001	2.02 (0.82)	2.98 (0.83)	<.001

a Comparison of scores of high, medium, and low achievers between the control and intervention groups.

b MCQ: multiple choice question.

c SAQ: short answer question.

d OSCE: objective structured clinical examination.

## Discussion

### Summary of Principal Findings

In this randomized controlled study of 690 first-year MBBS students, we found that e-modules, when added to traditional teaching, significantly improved cognitive, psychomotor, and affective domain scores compared with lectures alone, with the greatest gains seen among medium achievers. The study contributes to medical education literature by demonstrating how structured, theory-driven e-modules, explicitly designed for the Indian CBME environment, can support competency development at scale. While prior studies have examined e-learning in general, few have contextualized module design within India’s national curriculum reforms, digital readiness variations, and large cohort teaching realities. By aligning the e-modules with Bloom and Miller frameworks and evaluating them using the Kirkpatrick model, this study provides a comprehensive pedagogical and contextual analysis that is highly relevant for Indian medical colleges transitioning to CBME.

### Interpretation of Findings Using Educational Frameworks: Integrating Bloom Taxonomy and the Miller Pyramid in e-Module Design

The decision to implement the e-modules within first-year MBBS foundational subjects (anatomy, physiology, and biochemistry) was intentional, as these disciplines carry a high cognitive load and require extensive visualization and conceptual integration. Early exposure to structured, interactive digital learning in these subjects can strengthen foundational understanding, support the development of higher-order competencies, and better prepare students for subsequent clinical learning, where the CBME framework expects early competency formation.

The development and implementation of the e-module were carefully structured around Bloom taxonomy and the Miller pyramid to ensure a progressive, competency-based learning experience. These frameworks provided a solid foundation for designing digital learning tools that promote cognitive development and practical skill acquisition, aligning well with CBME.

To ensure a structured learning progression, Bloom taxonomy guided the organization of learning objectives within the module. This structured approach ensured that students moved beyond rote memorization [28] and developed deeper conceptual understanding and analytical skills. In addition to cognitive development, the Miller pyramid was integrated to ensure that learning translated into practical competence. By blending theoretical content with multimedia demonstrations and self-assessments, the module ensured that students were not only gaining theoretical knowledge but also demonstrating their ability to apply concepts in practical scenarios.

This study further evaluated the effectiveness of e-modules in medical education using the Kirkpatrick learning evaluation model, which assesses learning outcomes across 4 levels. The study specifically examined level 1 (reaction) and level 2 (learning), focusing on cognitive, affective (attitude), and psychomotor (skill) domains. e-Modules were found to significantly enhance student engagement, knowledge acquisition, skill performance, and professional attitudes in students, reinforcing their role as an effective supplement to traditional teaching methods.

### Level 1: Reaction—Student Perception of e-Modules

e-Modules effectively captured students’ interest and enhanced engagement, both of which are critical for promoting active learning. The use of interactive modules allows students to learn at their own pace, integrating clinical knowledge with foundational sciences, crucial for effective learning in medical education [29]. It has also been seen in previous works that integrated modular systems are more effective than traditional methods, promoting critical thinking and innovation among students [30]. These systems allow flexibility and adaptability to individual learning needs, which can lead to improved academic performance and satisfaction [30]. Students also expressed a strong preference for a blended learning approach, advocating for e-modules as a supplement rather than a replacement for traditional teaching.

### Level 2: Learning—Cognitive, Affective, and Psychomotor Outcomes

In terms of knowledge recall (remember), students in the intervention group were significantly better than those in the control group in recalling factual knowledge. However, low-achieving students did not show statistically significant improvements, likely due to difficulties in retaining information without contextual application. To enhance their learning, strategies, such as spaced repetition and adaptive quizzes, could be incorporated in e-modules to reinforce knowledge retention and provide targeted support [31].

For conceptual comprehension (understand), the e-module significantly improved comprehension for low and medium achievers, while high achievers showed minimal gains. This could be attributed to the fact that high-achieving students already have a strong conceptual foundation, making additional learning less impactful for them. To cater to this subgroup, future iterations of the module should integrate advanced case–based learning and real-world problem-solving tasks to challenge their cognitive abilities further [32]. When assessing problem-solving skills (apply), students in the intervention group showed significantly higher improvements. This finding underscores the module’s effectiveness in developing clinical reasoning and problem-solving abilities. Medium and low achievers benefited the most, suggesting that structured digital learning can help bridge performance gaps and create a more equitable learning experience. The effectiveness of e-modules in promoting critical thinking and higher-order learning (analyze) was evident through significant improvements in analysis-level assessments.

The impact of e-modules varied across different student groups. Medium achievers showed the most substantial gains across all cognitive levels. Their foundational knowledge gaps were likely addressed through the structured nature of the e-modules, allowing them to engage more effectively with new learning strategies. Low achievers demonstrated notable improvements in comprehension and application, indicating that e-modules serve as effective platforms for conceptual learning. In contrast, high achievers exhibited smaller relative gains, possibly due to their prior familiarity with the concepts covered. This finding aligns with past works, which also found that digital learning tools have the most impact on students with moderate baseline proficiency, while high-achieving students experience diminishing returns [33,34]. Students with moderate proficiency are often seen to benefit from digital learning tools as they enhance essential digital skills and cater to their specific learning needs, which are crucial for academic success [33,34].

Conversely, high-achieving students may already possess the skills and motivation to excel without the additional support of digital tools, potentially diminishing their relative impact [35,36]. Also, self-directed and problem-based learning approaches are more effective for high-achieving medical students, as they provide greater cognitive flexibility and autonomy compared to structured digital modules [37]. Studies have emphasized that digital learning tools designed for high achievers should incorporate more advanced problem-solving tasks, adaptive difficulty levels, and research-oriented challenges to maintain engagement and maximize learning outcomes [38-40].

### Effectiveness of e-Modules Across Progressive Levels (the Miller Pyramid)

This study’s findings align with the Miller framework, demonstrating that e-modules were particularly beneficial in progressing students from basic knowledge acquisition to higher-order thinking and practical application.

#### Knowledge Acquisition (Knows)—Remembering and Understanding

Students in the intervention group outperformed those in the control group in both of these domains. e-Modules enhanced students’ ability to recall and comprehend medical concepts more effectively than traditional lectures alone. This aligns with previous studies, where digital learning tools significantly improved retention and understanding in medical students by integrating multimedia, interactive elements, and adaptive learning [40,41].

Additionally, the effectiveness of e-learning in knowledge acquisition has been reported in studies on flipped classrooms [42]. Students who engaged with online modules before in-person sessions demonstrated better long-term retention compared to those relying solely on lectures.

#### Application of Knowledge (Knows How)—Application and Analysis

Low achievers and high achievers have performed significantly well in the intervention group, suggesting that e-modules facilitated deeper learning and cognitive processing, allowing students to synthesize and apply knowledge to real-world scenarios. It was similarly found in past studies that interactive e-learning modules significantly improved students’ problem-solving skills in clinical case studies [43-46]. Digital resources, when designed with active learning strategies, such as those integrated into these e-modules, enhance analytical reasoning, reduce cognitive overload [47], and improve knowledge retention and conceptual understanding [48,49].

#### Demonstration of Skills (Shows How)—Psychomotor Domain

The motor skills of students assessed using OSCEs were also significantly higher in the intervention group. The results suggest that interactive, visual, and simulation-based e-modules are effective in improving hands-on procedural skills, which is an essential aspect of medical training. The finding concurs with other studies that the use of computer technology enables learners to be active in the learning process, construct knowledge, develop problem-solving skills, and analyze the given situation to discover alternative solutions [50]. Studies have also found that virtual simulations and video-based learning improve students’ psychomotor skills, particularly in clinical scenarios where real-life practice opportunities are limited [51,52]. Simulation-based e-learning has been found as effective as traditional hands-on training in improving procedural skills [53]. Moreover, the integration of e-modules with interactive video demonstrations likely enhanced motor learning [54].

#### Professionalism and Attitudes (Does)—Affective Domain

The affective domain is often neglected in traditional didactic education. Studies suggest that interactive, self-paced learning methods enhance students’ motivation and self-directed learning behaviors [55], developing greater autonomy in medical education [56]. The incorporation of self-assessment, feedback, and real-life clinical scenarios in e-modules likely contributed to this improvement.

### Implications for CBME Implementation

The successful implementation of the e-module demonstrates that technology can enhance medical education by making complex concepts more accessible and promoting independent learning. One of the key takeaways is the role of technology as a catalyst for learning. The e-module supports self-directed learning by allowing students to engage with content at their own pace. This aligns with the growing emphasis on student autonomy and flexible learning within medical education [56]. Another critical aspect is the scalability and adaptability of the e-module. Its modular design allows for easy adaptation across various medical disciplines.

### Recommendations for Future Research

Future e-module designs should include differentiated instructions to address varied learning needs. CBME can benefit from hybrid models that combine e-modules with case-based learning and simulations, developing deeper engagement and critical thinking, particularly among high achievers. Adding gamified elements and competitive assessments may help sustain motivation across all student levels. Future research should extend evaluation to Kirkpatrick levels 3 and 4, examining behavior change during clinical postings and long-term impact on competency attainment in later MBBS phases. The integration of advanced adaptive learning, analytics dashboards, and gamification may further strengthen national-level CBME implementation.

### Limitations of the Study

This study has several limitations. The study was conducted between 2015 and 2018, and some technologies used (such as Adobe Flash) are now deprecated. While this does not affect the internal validity of the study, it limits immediate scalability of the exact modules in their original form. However, as all modules were exported into Moodle-compatible SCORM packages, the instructional design and learning architecture remain fully transferable to modern authoring tools. Additionally, the literature cited in this study spans both the study period and recent years, consistent with standard academic practice for studies reporting older datasets. Contemporary sources were intentionally included to ensure that the interpretation and implications of the findings align with current developments in CBME, e-learning research, and medical education frameworks. These later references did not inform the original study design or data collection but were used solely for contextualization. It involved only first-year medical students, limiting the generalizability of the findings. The focus was on short-term outcomes (Kirkpatrick levels 1 and 2), without examining long-term behavior changes or clinical impact. While most students had access to devices and the internet, differences in internet speed or device quality were not fully considered, which may have affected learning. Instructor-related bias is also possible, as differences in teaching style could have influenced results despite standardized content. Finally, while student feedback was gathered, detailed engagement data, such as time spent, interaction frequency, or assessment attempts, were not thoroughly analyzed.

### Conclusion

Well-designed e-modules, when combined with traditional teaching, can significantly enhance learning by using clear goals aligned with Bloom taxonomy and the Miller pyramid for undergraduate medical students. They helped students not only remember and understand content but also apply it and develop stronger attitudes and skills. Students across performance levels benefited, with average performers improving the most. Positive feedback and better test scores indicate that e-modules are a valuable addition to medical education, though they work best as supplements rather than replacements. These tools can be expanded to other subjects and learner levels to support more flexible and engaging learning. With CBME now uniformly implemented across India, scalable e-modules aligned with national competencies could promote more equitable learning, especially in resource-limited institutions. The findings support national strategies that use structured e-learning to strengthen and standardize instructional quality.

## Supplementary material

10.2196/84339 Multimedia Appendix 1 The screenshots of implementation of e-modules.

10.2196/84339 Multimedia Appendix 2 Sample of research randomizer results.

10.2196/84339 Multimedia Appendix 3 Knowledge, Attitude, and Practice towards e-learning.

10.2196/84339 Multimedia Appendix 4 List of e-modules addressing specific competency-based medical education (CBME) competencies and corresponding domains.

10.2196/84339 Multimedia Appendix 5 Overview of the development of e-modules.

10.2196/84339 Multimedia Appendix 6 Screenshot of steps of creating an e-module.

10.2196/84339 Multimedia Appendix 7 E module assessment questions.

10.2196/84339 Multimedia Appendix 8 Tool used for validation exercise.

10.2196/84339 Multimedia Appendix 9 Request for permission to use self-directed learning readiness (SDLR) scale and Visual, Aural, Read/Write, and Kinesthetic (VARK) questionnaire.

10.2196/84339 Multimedia Appendix 10 Feedback form for e-module.

10.2196/84339 Multimedia Appendix 11 Results of level 1 evaluation of the Kirkpatrick model.

10.2196/84339 Checklist 1 CONSORT checklist.

## Abbreviations

CBME: competency-based medical education
EBIA: end-of-block internal assessment
MCQ: multiple choice question
Moodle-SCORM: Modular Object-Oriented Dynamic Learning Environment
