# Supplementary material for: E-Learning Modules Based on Bloom Taxonomy and the Miller Pyramid for First-Year Indian Medical Students: Randomized Controlled Study in Medical Education
Source: JMIR Hum Factors. 2026 Apr 7;13:e84339. doi: 10.2196/84339 (PMC13055945; doi:10.2196/84339)

Supplementary file 7

The screenshots of implementation of e-modules through SRU MOODLE

Figure 1: Homepage of SRU MOODLE website at the beginning of the study: www.eli.com

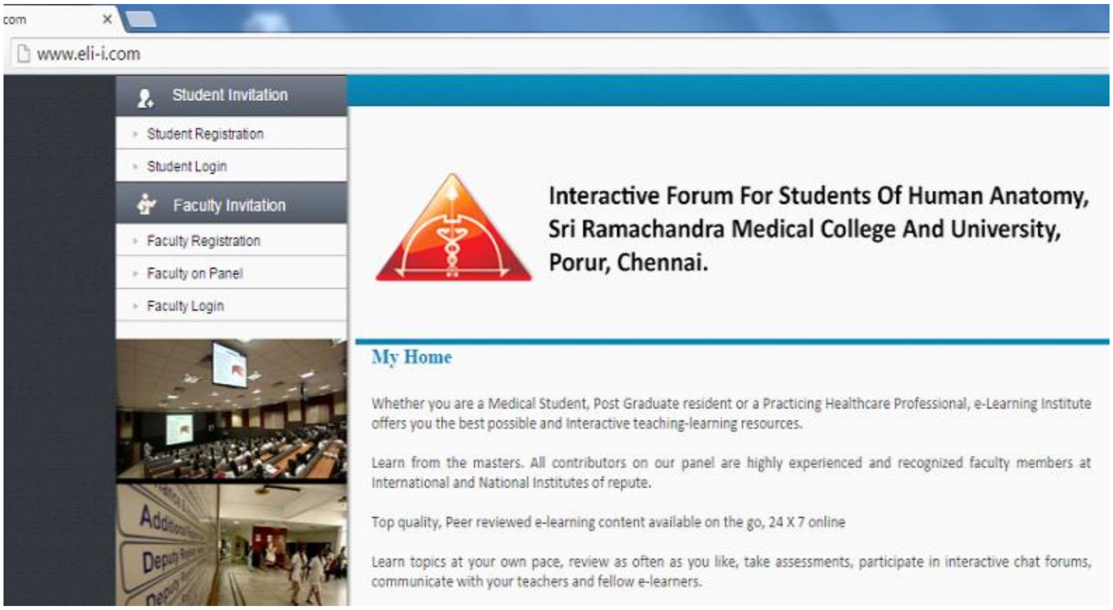

Figure 2: Registration page at the beginning of the study

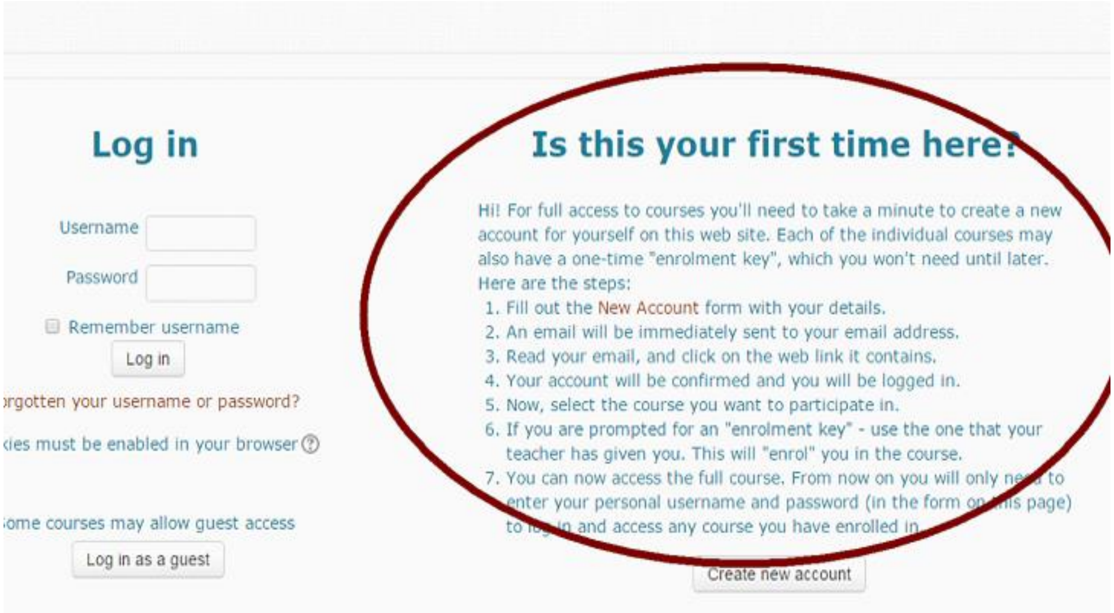

Supplementary file 7

The screenshots of implementation of e-modules through SRU MOODLE

Figure 3: Homepage after upgrading SRU MOODLE site http://elearning.sriramachandra.edu.in/

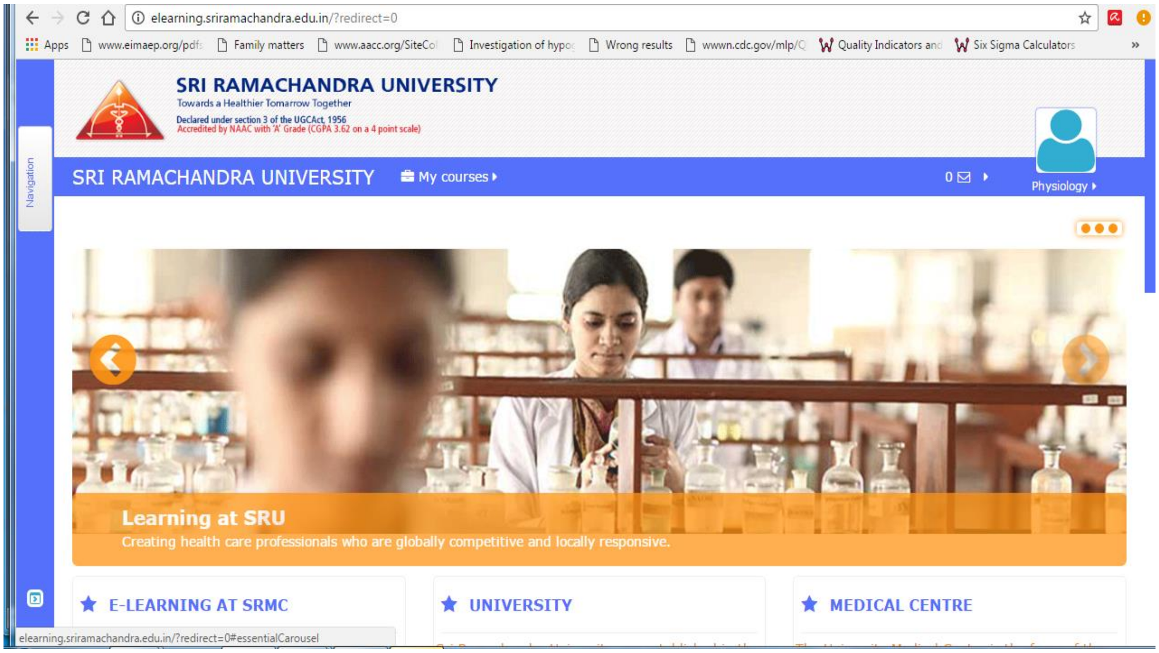

Figure 4: Registration page after upgrading SRU MOODLE site

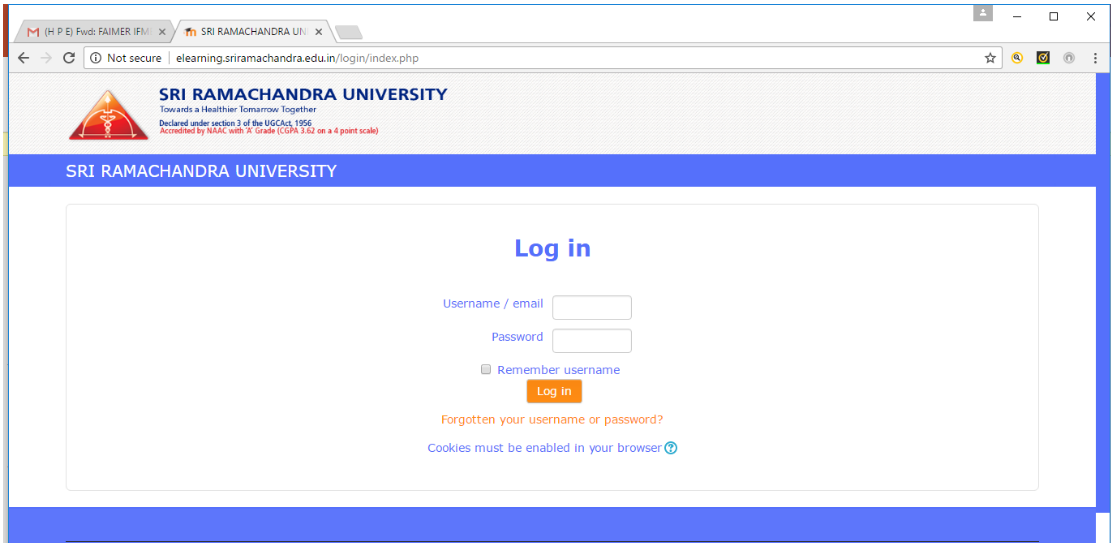

Supplementary file 7

The screenshots of implementation of e-modules through SRU MOODLE

Figure 5: Screenshot showing the various departments in SRU MOODLE

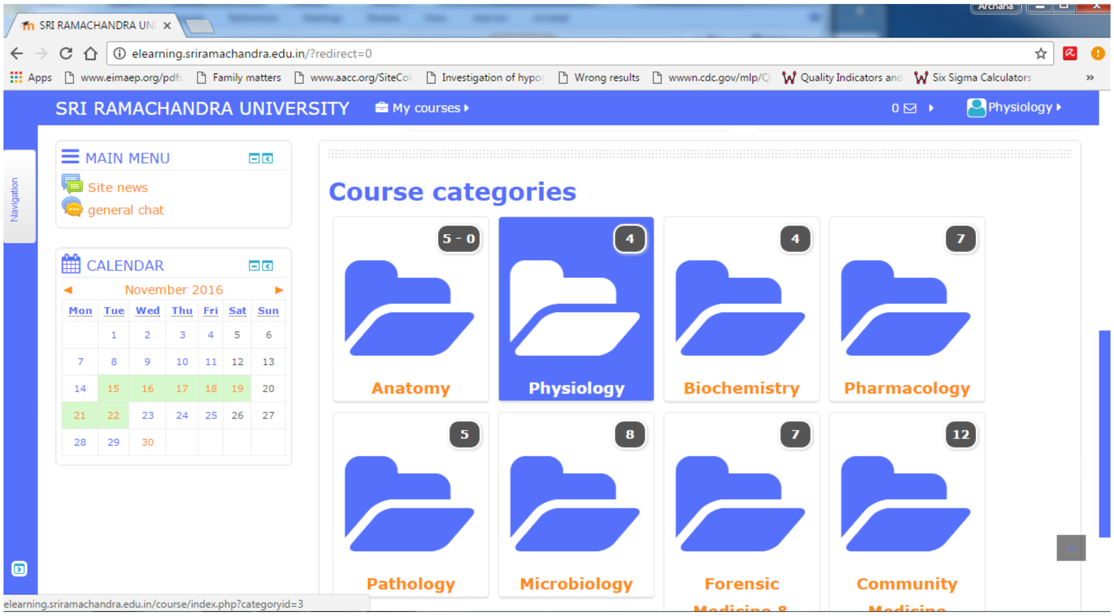

Figure 6: Screenshot showing the Physiology admin page in SRU MOODLE

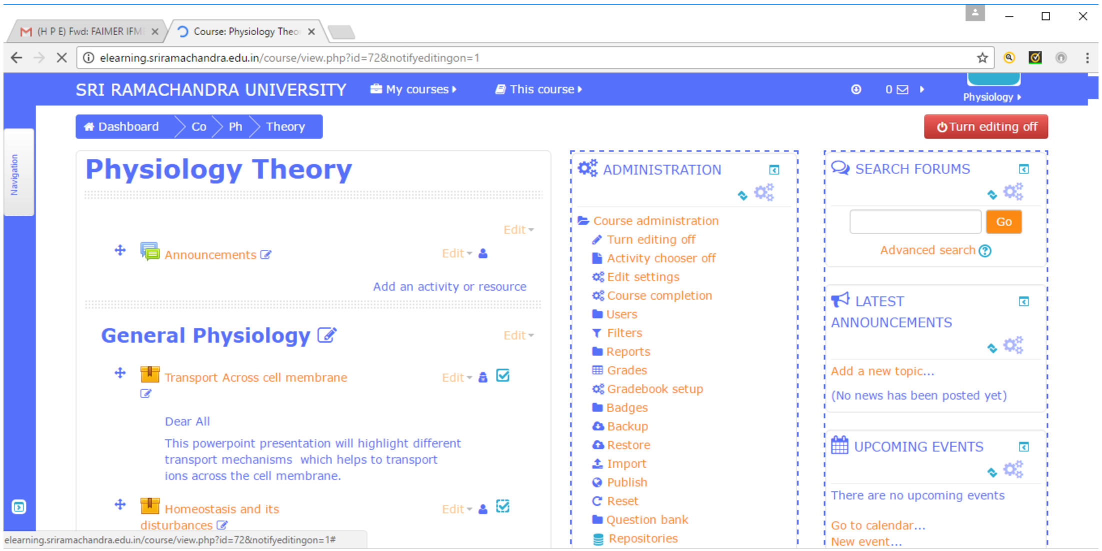

Supplement: Multimedia Appendix 1 [file humanfactors-v13-e84339-s001.pdf]
