# Supplementary material for: E-Learning Modules Based on Bloom Taxonomy and the Miller Pyramid for First-Year Indian Medical Students: Randomized Controlled Study in Medical Education
Source: JMIR Hum Factors. 2026 Apr 7;13:e84339. doi: 10.2196/84339 (PMC13055945; doi:10.2196/84339)
Supplement: Multimedia Appendix 2 [file humanfactors-v13-e84339-s002.pdf]

Research Randomizer Results

2 Sets of 125 Unique Numbers Per Set
Range: From 1 to 250 -- Sorted from Least to Greatest

Job Status: Working... Please be patient...

Set #1:

4, 6, 7, 8, 11, 12, 13, 16, 17, 21, 23, 25, 26, 27, 28, 30, 32, 33, 37, 39, 43, 44, 45, 47, 48, 50, 51, 52, 53, 62, 64, 65, 66, 70, 71, 73, 74, 77, 78, 80, 81, 82, 84, 88, 91, 92, 95, 96, 97, 102, 103, 104, 105, 107, 108, 111, 112, 113, 114, 116, 118, 119, 120, 122, 127, 129, 131, 136, 140, 141, 144, 145, 147, 149, 151, 153, 154, 156, 157, 158, 159, 161, 162, 163, 164, 165, 167, 170, 172, 173, 175, 176, 180, 181, 182, 183, 188, 190, 193, 194, 198, 201, 203, 204, 205, 206, 207, 209, 212, 215, 217, 219, 224, 226, 227, 231, 232, 233, 235, 238, 242, 245, 247, 248, 250

Set #2:

1, 3, 4, 6, 8, 10, 11, 13, 14, 15, 18, 19, 25, 28, 29, 30, 31, 32, 34, 36, 39, 40, 42, 43, 46, 52, 53, 55, 60, 62, 69, 72, 75, 76, 79, 80, 82, 84, 87, 89, 91, 94, 95, 98, 104, 105, 109, 114, 115, 120, 121, 122, 124, 126, 131, 133, 134, 135, 138, 139, 140, 141, 145, 147, 148, 149, 151, 153, 154, 155, 156, 157, 158, 160, 161, 162, 164, 167, 169, 172, 173, 174, 175, 176, 177, 178, 179, 180, 181, 182, 183, 185, 187, 190, 191, 192, 195, 196, 199, 203, 204, 206, 211, 212, 213, 214, 217, 218, 221, 222, 223, 225, 226, 231, 233, 234, 235, 236, 237, 238, 239, 242, 243, 246, 248
