# Supplementary material for: E-Learning Modules Based on Bloom Taxonomy and the Miller Pyramid for First-Year Indian Medical Students: Randomized Controlled Study in Medical Education
Source: JMIR Hum Factors. 2026 Apr 7;13:e84339. doi: 10.2196/84339 (PMC13055945; doi:10.2196/84339)
Supplement: Multimedia Appendix 3 [file humanfactors-v13-e84339-s003.pdf]

Knowledge, Attitude and Practice towards e - Learning among

Undergraduate Health Professions Students

A: General Information

Age: _____ years

Gender: ☐1 Male ☐2 Female

Have you undergone formal computer training earlier? ☐1 yes ☐2 No

B: Availability of computers and internet connectivity (AVL):

AVL1. Where do you have access to internet? ☐1At college ☐2At home ☐3 Internet cafe ☐4 Personal data card

AVL2. Do you possess: ☐1 desktop ☐2 laptop ☐3 Palmtop/PDA ☐4 Others, please specify ____________ ☐5 None

AVL3. How would you describe your access to computers?

☐1very poor ☐2 poor ☐3 adequate ☐4Good ☐5very good

AVL4. Average time spent on internet:

☐1< 1 hour/day ☐2 1 to 2 hours /day ☐32 to 3 hours/day ☐4 3 to 4 hours/day ☐5>4 hours/day

AVL5. Frequency of internet use:

☐1 never ☐2 1 to 2 times / 6 months ☐3 1 to 2 times / month ☐4 1 to 2 times / week ☐5Daily

AVL6. How would you describe your access to internet connectivity?

☐1very poor ☐2 poor ☐3 adequate ☐4Good ☐5very good

C: How often do you use internet for the following purposes (PUR)?

Please select based on the scale given below:
1 - Never, 2 - Occasionally, 3 - Sometimes, 4 - Most of the times, 5 – Always

Purpose		1	2	3	4	5
PUR1.	General browsing (e.g.: news, sports etc)					
PUR2.	Communication (e.g.: Email, chat etc)					
PUR3.	Entertainment (e.g.: Games, music etc)					
PUR4.	Online transaction					
PUR5.	Therapeutic guidelines					
PUR6.	Recent advances in healthcare					

D: How do you describe your confidence level while performing the following tasks (CON)?

Please select based on the scale given below:
1 - Never, 2 - Occasionally, 3 - Sometimes, 4 - Most of the times, 5 - Always

Tasks		1	2	3	4	5
CON1.	Using a word document					
CON2.	Creating a power point presentation					
CON3.	Sending email with attachments					
CON4.	Analysing data with statistics software					
CON5.	Creating computerized patient record					
CON6.	Creating a web page / blog					
CON7.	Downloading relevant information from internet					
CON8.	Using an excel spreadsheet					
CON9.	Writing a computer program					
CON10.	Drawing using ‘paint’					

Knowledge, Attitude and Practice towards e - Learning among

Undergraduate Health Professions Students

E. How often do you prefer the following resources for studying purposes (PRE)?
Please select based on the scale given below:
1 - Never, 2 - Occasionally, 3 - Sometimes, 4 - Most of the times, 5 - Always

Resources		1	2	3	4	5
PRE1.	CD-ROMs					
PRE2.	Journals					
PRE3.	Lecture notes					
PRE4.	Textbooks					
PRE5.	Internet					

Others* (Please specify) ---------------

F. To what extent do you agree or disagree with the following features as enhancer of your e - learning experience (LEX)
Please select based on the scale given below:
1 - Strongly Disagree, 2 - Disagree, 3 - Neutral, 4 - Agree, 5 - Strongly Agree

Features		1	2	3	4	5
LEX1.	Simple / self-explanatory images					
LEX2.	Web-based power point / textbooks					
LEX3.	Teaching through Learning management systems e.g.: Moodle					
LEX4.	Animations					
LEX5.	Video and audio					
LEX6.	Virtual simulators to learn clinical skills					
LEX7.	Online assignment / Quiz					
LEX8.	Online discussion with faculty					

G. To what extent do you agree or disagree with the following as advantages of e-learning (PAE)
Please select based on the scale given below:
1 - Strongly Disagree, 2 - Disagree, 3 - Neutral, 4 - Agree, 5 - Strongly Agree

		1	2	3	4	5
PAE1.	Time saving					
PAE2.	Nothing specific					
PAE3.	Available anywhere / any time					
PAE4.	Scope for self-assessment					
PAE5.	More interactive					
PAE6.	Updated information					

Others* (Please specify) ---------------

Knowledge, Attitude and Practice towards e - Learning among

Undergraduate Health Professions Students

H. To what extent do you agree or disagree with the following as limitations of e-learning (PLE)

Please select based on the scale given below:
1 - Strongly Disagree, 2 - Disagree, 3 - Neutral, 4 - Agree, 5 - Strongly Agree

		**1**	**2**	**3**	**4**	**5**
PLE1.	Requires computer skills					
PLE2.	Availability of internet connectivity					
PLE3.	Availability of computers					
PLE4.	Time consuming					
PLE5.	Distractions due to other non – academic activities					
PLE6.	Absence of human element					
PLE7.	Requires self-motivation					
PLE8.	Excess of unwanted information					
PLE9.	Computer screen hard to focus for a long time					
PLE10.	Authenticity of available information					
PLE11.	Not part of regular curriculum					

Others* (Please specify) ---------------

I: To what extent do you agree or disagree with the following as educational impact of e-learning (PEI)

Please select based on the scale given below:
1 - Strongly Disagree, 2 - Disagree, 3 - Neutral, 4 - Agree, 5 - Strongly Agree

**Educational impact**		**1**	**2**	**3**	**4**	**5**
PEI1.	It facilitates both teaching & learning					
PEI2.	It helps in long term retention of content					
PEI3.	It is a supplementary tool in addition to regular classroom sessions					
PEI4.	It is vital for acquiring more competency					
PEI5.	It should replace all lectures / labs					
PEI6.	It helps in better comprehension of difficult topics					
PEI7.	Computer and internet use should be encouraged in teaching institutions					

Others* (Please specify) ---------------

J. What do you think as the **major advantage** of introducing e – learning in medical/ dental education in India?

K. What do you think as the **major disadvantage** of introducing e – learning in medical/ dental education in India?

L. What do you think as the **biggest challenge** in introducing e – learning in medical/ dental education in India?
