# Supplementary material for: E-Learning Modules Based on Bloom Taxonomy and the Miller Pyramid for First-Year Indian Medical Students: Randomized Controlled Study in Medical Education
Source: JMIR Hum Factors. 2026 Apr 7;13:e84339. doi: 10.2196/84339 (PMC13055945; doi:10.2196/84339)
Supplement: Multimedia Appendix 4 [file humanfactors-v13-e84339-s004.pdf]

Supplementary file 2

(i) List of e-modules addressing specific CBME competencies and corresponding domains

Name of the e-modules	MCI CBME# Competency	Competencies	Domain K/S/A/C*	Core Y/N**
High altitude physiology (Pilot study)	PY6.4	Describe and discuss the physiology of high altitude	K	Y
Integrated e-module on diabetes mellitus	PH1.36	Describe the mechanism of action, types, doses, side effects, indications and contraindications of drugs used in diabetes mellitus	. K	Y
	AN52.1	Describe & identify the microanatomical features of the Pancreas	K	Y
	PY8.2	Describe the synthesis, secretion, transport, physiological actions, regulation and effect of hypo secretion of the pancreas	K	Y
	BI3.4	Define and differentiate the pathways of carbohydrate metabolism (glycolysis, gluconeogenesis, glycogen metabolism)	K	Y
	BI3.5	Describe and discuss the regulation, functions and integration of carbohydrate along with associated diseases/disorders	K	Y
	BI3.9	Discuss the mechanism and significance of blood glucose regulation in health and disease	K	Y
	PA32.4	Classify and describe the epidemiology, etiology, pathogenesis, pathology, clinical laboratory features, complications and progression of diabetes mellitus	K	Y
	BI11.17	Explain the basis and rationale of biochemical tests done in the following conditions: diabetes mellitus	K	Y
	BI3.8	Discuss and interpret laboratory results of analytes associated with the metabolism of carbohydrates	K/S	Y
	BI3.10	Interpret the results of blood glucose levels and other laboratory investigations related to disorders of carbohydrate metabolism	K/S	Y
Juxta Glomerular Apparatus (JGA)	PY7.2	Describe the structure and functions of the juxta-glomerular apparatus and the role of the renin-angiotensin system	K	Y
Peak Expiratory Flow Rate (PEFR)	CT2.11	Describe, discuss and interpret pulmonary function tests	K/S/A/C	Y

#Medica Council of India – Competency Based Medical Education, *K-Knowledge, S- Skill, A – Attitude, C-Communication, **Y- Yes, N-No

Supplementary file 2

(ii) E-module on Diabetes Mellitus - list of objectives classified based on CBME Competency, Bloom's taxonomy and Miller's pyramid

Integrated e-module on Diabetes Mellitus				
**SLO Code**	**Specific Learning objectives (SLO)**	**CBME Competency**	**Taxonomy Bloom's level**	**Taxonomy Miller's Pyramid**
DM01	List the hormones secreted by alpha, beta, and delta cells	PY8.2	Remember	Knows
DM02	List the physiological actions of insulin	PY8.2		
DM03	List the physiological actions of glucagon	PY8.2		
DM04	State the Normal blood glucose concentration in blood	PY8.2		
DM05	Define Type 1 Diabetes	PA32.4		
DM06	Define Type 2 Diabetes	PA32.4		
DM07	Describe the gross and microanatomy of the Pancreas	AN52.1	Understand	Knows How
DM08	Describe the structure, synthesis and release of Insulin	PY8.2		
DM09	Describe the steps in synthesis and release of glucagon	PY8.2		
DM10	Explain the mechanism of action of insulin	PY8.2		
DM11	Explain the mechanism of action of glucagon	PY8.2		
DM12	Describe the metabolic effects of insulin and glucagon (on carbohydrate, protein and fat metabolism)	BI3.4		
DM13	Explain the mechanism of blood glucose regulation in health and disease	BI3.5 BI3.9		

Supplementary file 2

DM14	Choose the appropriate investigations for type 1 and type 2 DM (Urinary Glucose, Fasting and post prandial Blood Glucose, Insulin Concentrations, Glycated Hemoglobin, Glucose Tolerance Test)	BI11.17	Apply	Knows How
DM15	Interpret the lab investigations related to DM	BI3.10<br>BI3.8		
DM16	Differentiate between type 1 and type 2 DM	PA32.4		
DM17	Compare and contrast the metabolic effects of insulin and glucagon on carbohydrate, protein and fat metabolism	PY8.2	Analyze	Shows How
DM18	Relate pathophysiology of DM to cardinal symptoms (Polydipsia, Polyuria, Polyphagia) and complications (neuropathy, nephropathy, retinopathy etc) of DM	PA32.4		
DM19	Outline the management of type 1 and type 2 DM (Insulin, Weight loss, bariatric surgery, thiazolidinediones, metformin, sulfonylureas etc)	PH1.36		

Supplementary file 2

(iii) E-module on JGA - List of objectives classified based on CBME Competency, Bloom's taxonomy and Miller's pyramid

**E module on Juxta Glomerular Apparatus (JGA)**				
**SLO Code**	**Specific Learning objectives (SLO)**	**CBME Competency**	**Taxonomy Bloom's level**	**Taxonomy Miller's Pyramid**
JG01	State the normal Glomerular Filtration Rate (GFR)	PY7.2	Remember	Knows
JG02	State the normal Renal Blood Flow (RBF)	PY7.2		
JG03	Name the cells in Juxta Glomerular Apparatus (JGA)	PY7.2		
JG04	Identify the cells in JGA	PY7.2		
JG05	Describe the structure of the JGA	PY7.2	Understand	Knows How
JG06	Describe the functions of Macula Densa cells	PY7.2		
JG07	Describe the functions of Juxta Glomerular cells	PY7.2		
JG08	Describe the functions of Extraglomerular mesangial cells (Lacis cells)	PY7.2		
JG09	Examine the role of macula densa in regulating RBF & GFR	PY7.2	Apply	Knows How
JG10	Relate the role Renin - Angiotensin II mechanism to autoregulation of RBF and GFR	PY7.2		
JG11	Compare and contrast Angiotensin I and Angiotensin II	PY7.2	Analyze	Shows How
JG12	Analyze the role of Tubulo-Glomerular Feedback (TGF) in autoregulation of RBF and GFR	PY7.2		

Supplementary file 2

(iv) E-module on HAP - List of objectives classified based on CBME Competency, Bloom's taxonomy and Miller's pyramid

E module on High Altitude Physiology (HAP - Pilot Study)				
**SLO Code**	**Specific Learning objectives (SLO)**	**CBME Competency**	**Taxonomy Bloom's level**	**Taxonomy Miller's Pyramid**
HA01	State the normal barometric pressure at sea level	PY6.4	Remember	Knows
HA02	List the acute effects of hypoxia	PY6.4		
HA03	Describe the effect of oxygen at different altitudes on saturation of hemoglobin	PY6.4	Understand	Knows How
HA04	Describe in detail acclimatization to hypoxia	PY6.4		
HA05	Explain cellular acclimatization	PY6.4		
HA06	Describe chronic mountain sickness	PY6.4		
HA07	Explain pathophysiology of High-Altitude Pulmonary Edema (HAPE)	PY6.4	Apply	Knows How
HA08	Explain pathophysiology of High-Altitude Cerebral Edema (HACE)	PY6.4		
HA09	Outline management of acute mountain sickness	PY6.4		

Supplementary file 2

(v) E-module on PEFR - List of objectives classified based on CBME Competency, Bloom's taxonomy and Miller's pyramid

**E module on Measurement of Peak Expiratory Flow Rate (PEFR)**					
**SLO Code**	**Specific Learning objectives (SLO)**	**CBME Competency**	**Domain**	**Taxonomy Bloom's level**	**Taxonomy Miller's Pyramid**
PE01	State the normal PEFR value	CT2.11	K	Remember	Knows
PE02	List the indications for PEFR	CT2.11	K	Remember	Knows
PE03	List the contraindications for PEFR	CT2.11	K	Remember	Knows
PE04	Describe the importance of measuring PEFR	CT2.11	K	Understand	Knows How
PE05	Explain the clinical applications of PEFR	CT2.11	K	Apply	Knows How
PE06	Greet the subject / Standardized Patient (SP)	CT2.11	A/C	Responding	Shows How
PE07	Explain the procedure of measuring PEFR to the subject	CT2.11	S	Manipulation*	Shows How
PE08	Obtain the informed consent	CT2.11	A/C	Responding	Shows How
PE09	Measure PEFR of the subject, following all the steps appropriately	CT2.11	S	Manipulation*	Shows How
PE10	Repeat the above procure three times	CT2.11	S	Manipulation*	Shows How
PE11	Interpret the measured values	CT2.11	K	Analyze	Shows How
PE12	Thank the subject	CT2.11	A	Responding	Shows How

*Classification by Dave, 1970

Supplementary file 2

(vi) List of assessment tools for E-modules classified based on CBME Competency, Bloom's taxonomy and Miller's pyramid

SLO Code#	CBME competency no.	Domain	Taxonomy Bloom's level	Taxonomy Miller's Pyramid	Assessment tool used in e-module	Assessment tool used in end of block internal assessment**
	Integrated e-module on Diabetes Mellitus					
DM01<br>DM02<br>DM03<br>DM04<br>DM05<br>DM06	PY8.2, PA32.4, PA32.4	Knowledge	Remember	Knows	MCQs, Match the following, hot spot (for identifying the structure)	MCQs
DM07<br>DM08<br>DM09<br>DM10<br>DM11<br>DM12<br>DM13	AN52.1, PY8.2, BI3.4, BI3.5, BI3.9	Knowledge	Understand / comprehension	Knows How	MCQs, drag and drop, true or false, choose all the correct options (multiple options)	Problem based MCQs
DM14<br>DM15<br>DM16	BI11.17, BI3.10, BI3.8, PA32.4	Knowledge	Apply	Knows How	Problem based MCQs	Problem based MCQs
DM17<br>DM18<br>DM19	PY8.2, PA32.4, PH1.36	Knowledge	Analyze	Shows How	Problem based MCQs	Short Notes

Supplementary file 2

	E module on Juxta Glomerular Apparatus					
JG01<br>JG02<br>JG03<br>JG04	PY7.2	Knowledge	Remember	Knows	MCQs	MCQs
JG05<br>JG06<br>JG07<br>JG08	PY7.2	Knowledge	Understand	Knows How	MCQs, true or false	Problem based MCQs
JG09<br>JG10	PY7.2	Knowledge	Apply	Knows How	Problem based MCQs	Problem based MCQs
JG11<br>JG12	PY7.2	Knowledge	Analyze	Shows How	Problem based MCQs	Short Notes
	E module on Measurement of Peak Expiratory Flow Rate (PEFR)					
PE01	CT2.11	Knowledge	Remember	Knows	MCQs	MCQs
PE02	CT2.11	Knowledge	Remember	Knows		
PE03	CT2.11	Knowledge	Remember	Knows		
PE04	CT2.11	Knowledge	Understand	Knows How		
PE05	CT2.11	Knowledge	Apply	Knows How		Problem based MCQs
PE06	CT2.11	Attitude	Responding	Shows How	MCQs based on simulated case scenarios	OSCE
PE07	CT2.11	Skill	Manipulation*	Shows How		OSCE
PE08	CT2.11	Attitude	Responding	Shows How		OSCE

Supplementary file 2

PE09	CT2.11	Skill	Manipulation*	Shows How		OSCE
PE10	CT2.11	Skill	Manipulation*	Shows How		OSCE
PE11	CT2.11	Knowledge	Analyze	Shows How	Problem based MCQs	
PE12	CT2.11	Attitude	Responding	Shows How		OSCE

#Detailed list of SLOs are shown in tables i to iv
*Classification by Dave, 1970
**SRIHER follows block system of assessment for the undergraduate curriculum
