# Supplementary material for: E-Learning Modules Based on Bloom Taxonomy and the Miller Pyramid for First-Year Indian Medical Students: Randomized Controlled Study in Medical Education
Source: JMIR Hum Factors. 2026 Apr 7;13:e84339. doi: 10.2196/84339 (PMC13055945; doi:10.2196/84339)
Supplement: Multimedia Appendix 5 [file humanfactors-v13-e84339-s005.pdf]

Development of E-modules

E-modules were developed as interactive learning tools.

The development process included:

a) Content Creation: Text, images and animations were designed by subject matter experts, IT professionals and educational specialists. Images were sourced from textbooks and public domain resources under fair use policies (U.S. Copyright Office Fair Use Index, 2013)[24]. Missing illustrations were manually created using Adobe Flash, Microsoft Paint and Photoshop. Brief explanations and relevant information required for understanding the important concepts were drafted using simple sentences in English. Many of the self-assessment questions were developed by experts, while some of them were taken from standard question banks and acknowledged by quoting the references.

b) Conversion of images into animation: The images were broken down into several components and effectively animated using Adobe Flash Player.

c) Recording and editing of audio: The audio was recorded in the e-learning studio of the host institution and edited using Audacity software version 2.1.2.

d) Adding text and audio to the animation: The texts were added sequentially and care was taken to ensure that the audio and video were matched properly. Videos were captured using a Sony Handycam DCR-SX40 and subsequently edited with Adobe Premiere Pro CS6.

e) Addition of interactivity: The e-modules were mad interactive by using software called ‘Knowledge Presenter X’(KpX) (Knowledge Presenter X, 2010). KpX is a program that allows users to import photographs, images, text, documents and/or multimedia, synchronize them on a timeline or to steps and create a file that can be played in almost any browser, whether over the Internet, Intranet, from local disk or CD-ROM.

f) Interactive self-assessment: Each module included 10–20 self-assessment questions (MCQs, case-based questions and concept-based questions) with built-in scoring and instant feedback. No restrictions were placed on reattempts. The reporting of results with scoring was displayed at the end of each assessment module.

C). Development of PowerPoint: Identical content (texts, images) was used in the PowerPoint presentation and e-modules to rule out any content bias during the process of implementation.
