# Supplementary material for: E-Learning Modules Based on Bloom Taxonomy and the Miller Pyramid for First-Year Indian Medical Students: Randomized Controlled Study in Medical Education
Source: JMIR Hum Factors. 2026 Apr 7;13:e84339. doi: 10.2196/84339 (PMC13055945; doi:10.2196/84339)

Supplementary file 3

Screenshot of steps of creating an e -module using rapid authoring tool (Knowledge presenter X)

Figure 1: Start up screen of Knowledge presenter X (KpX)

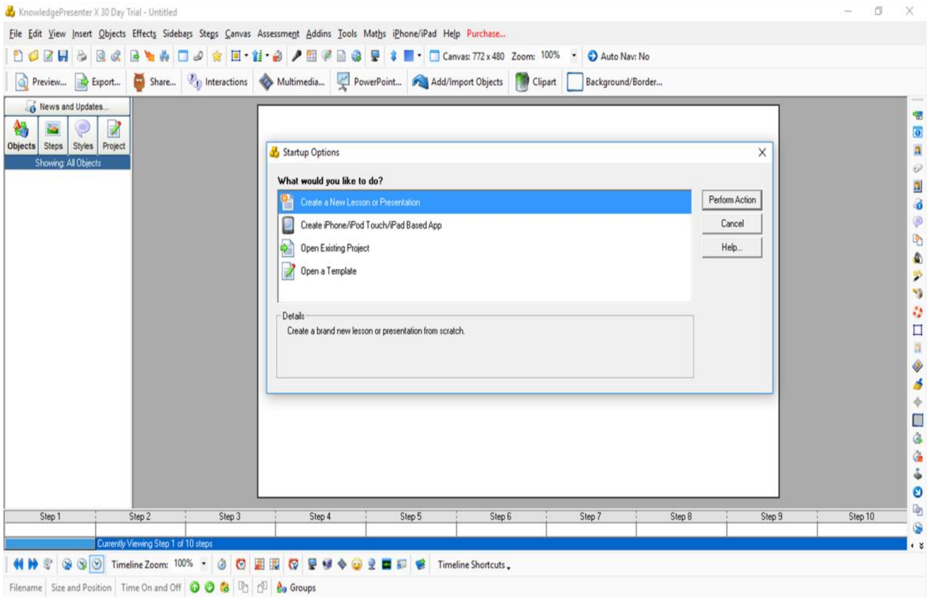

Figure 2: Interface of KpX showing various options in lessons creation

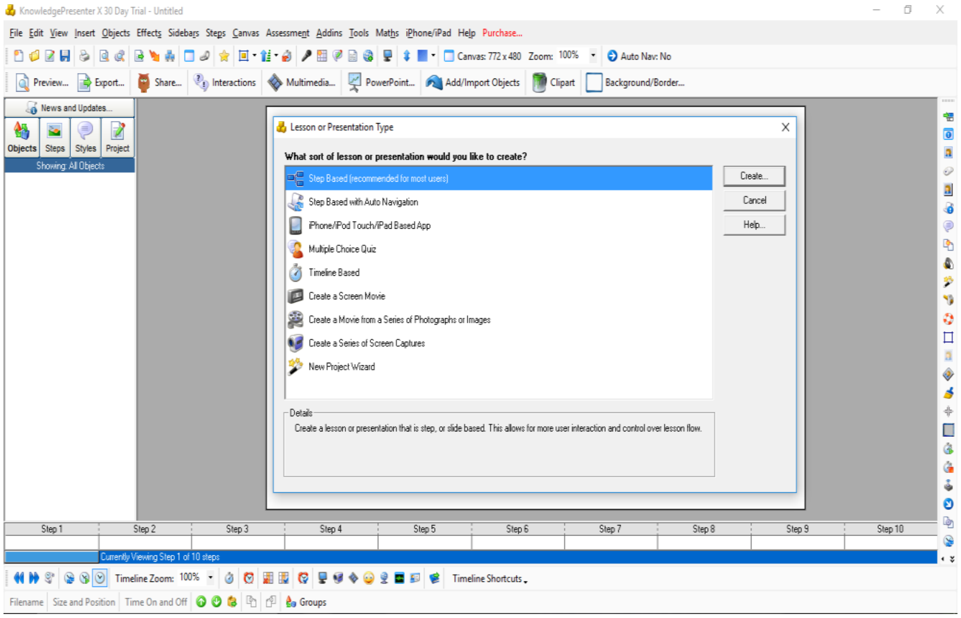

Supplementary file 3

Screenshot of steps of creating an e -module using rapid authoring tool (Knowledge presenter X)

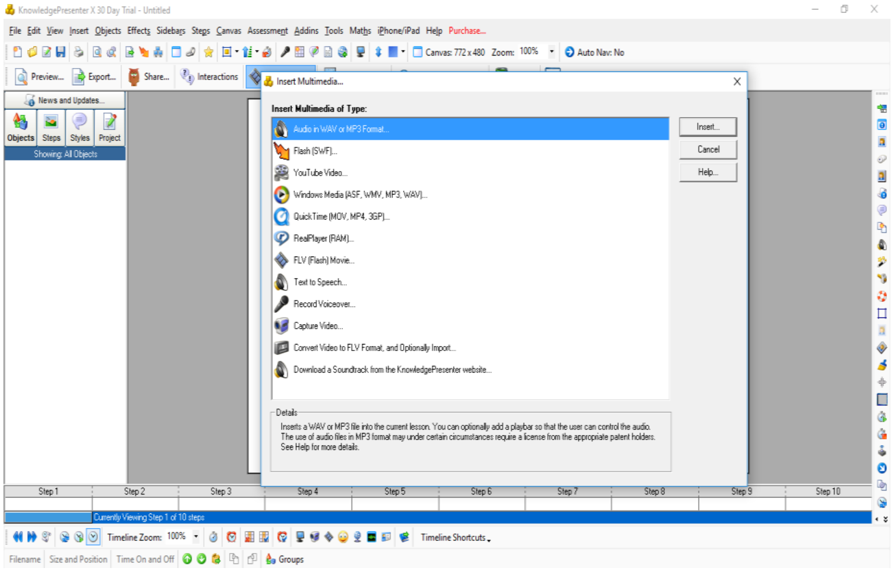

Figure 3: Interface of KpX showing various options in multimedia

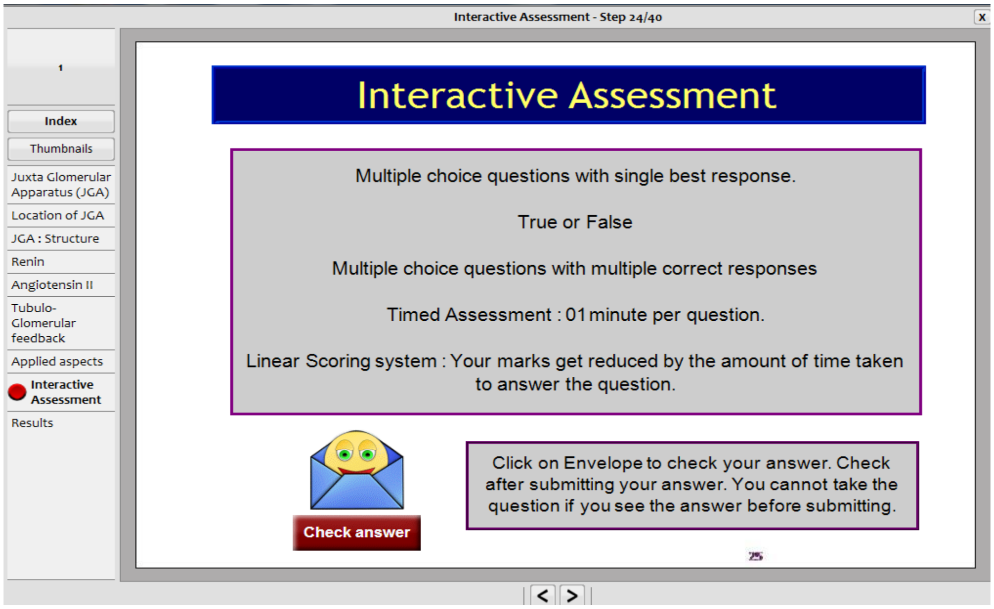

Figure 4: Interface of KpX showing general instructions about assessment

Supplementary file 3

Screenshot of steps of creating an e -module using rapid authoring tool (Knowledge presenter X)

Figure 5: Interface of KpX showing Multiple Choice question with single best response

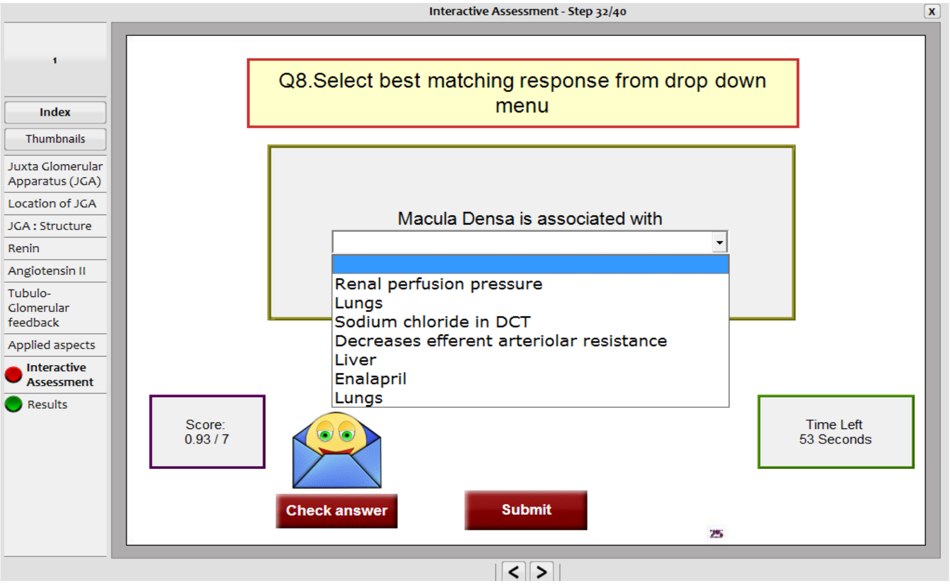

Figure 6: Interface of KpX showing MCQs with multiple correct responses

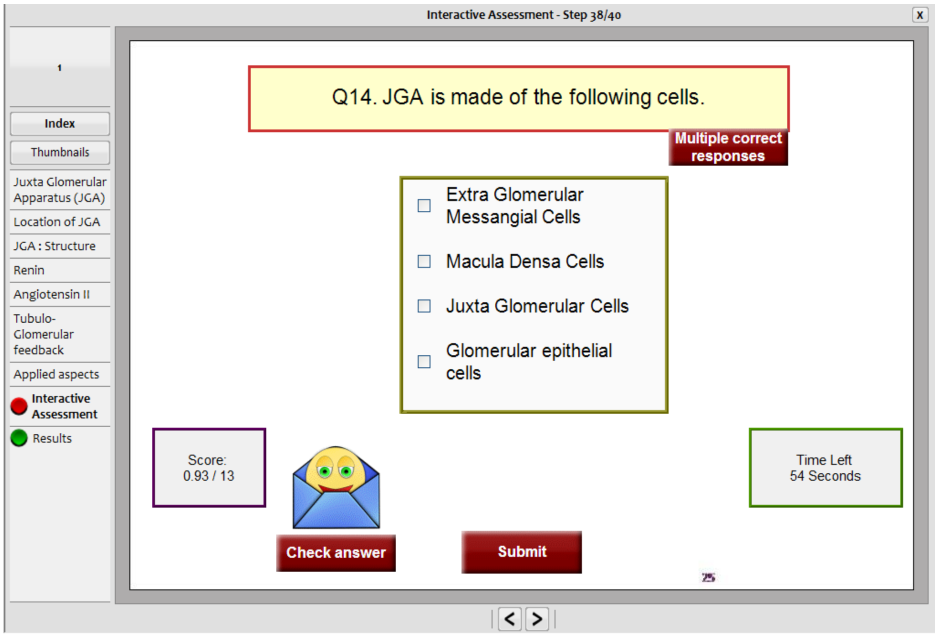

Supplementary file 3

Screenshot of steps of creating an e -module using rapid authoring tool (Knowledge presenter X)

Figure 7: Interface of KpX showing the reporting of results

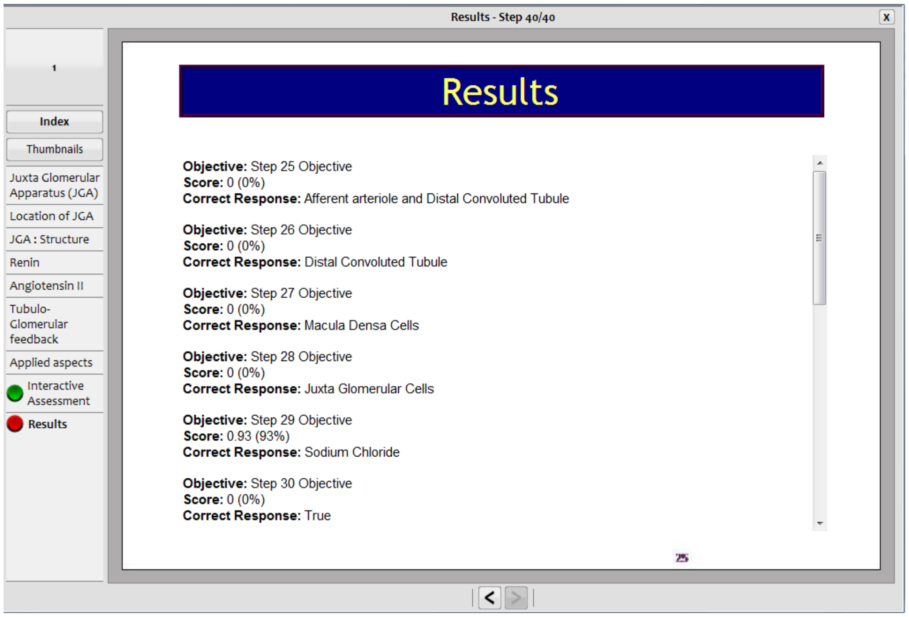

Supplement: Multimedia Appendix 6 [file humanfactors-v13-e84339-s006.pdf]
