# Supplementary material for: E-Learning Modules Based on Bloom Taxonomy and the Miller Pyramid for First-Year Indian Medical Students: Randomized Controlled Study in Medical Education
Source: JMIR Hum Factors. 2026 Apr 7;13:e84339. doi: 10.2196/84339 (PMC13055945; doi:10.2196/84339)
Supplement: Multimedia Appendix 7 [file humanfactors-v13-e84339-s007.pdf]

e-module assessment questions

Topic: 'Functions of Insulin and Glucagon'

Multiple choice questions

Questions 1- 8 have single BEST response

Please encircle the correct answer

1. Glucagon is secreted by
   a. PP cells
   b. A cells or alpha cells
   c. B cells or beta cells
   d. D cells or delta cells

2. C peptide concentration in blood provides an index of
   a. alpha cell function
   b. beta cell function
   c. PP cell function
   d. d.delta cell function

3. A chain and B chain of Insulin are connected by
   a. one Disulphide bridges
   b. two Disulphide bridges
   c. three Disulphide bridges
   d. four Disulphide bridges

4. The secretion of insulin is stimulated by the following EXCEPT
   a. Fatty acids
   b. Amino acids
   c. Glucagon
   d. Adrenaline

5. Insulin has following effect on carbohydrate metabolism
   a. Stimulates glycogenolysis in liver &muscle
   b. Stimulates gluconeogenesis in the liver
   c. Inhibits peripheral utilization of glucose
   d. Promotes glycogenesis in liver &muscle

6. Insulin was first isolated by
   a. Sanger
   b. Claude bernard
   c. Watson & Crick
   d. Banting & Best

7. One of the following hormone is an anabolic hormone.
   a. Glucagon
   b. Parathormone
   c. Insulin
   d. Cortisol

8. WHO (World Health Organisation) criteria for normal fasting plasma glucose is
   a. < 100 mg/dl
   b. < 110 mg/dl
   c. < 120 mg/dl
   d. < 130 mg/dl

9. The following statements represent various steps involved in Insulin secretion.
   1. Phosphorylation of beta subunit
   2. Binding of alpha unit of transmembrane receptor
   3. Insertion of glucose transporters
   4. Generation of Insulin Reaction Substrates

Choose the appropriate order of sequence from the options given below
   a. 1,2,4,3
   b. 2,1,4,3
   c. 3,1,4,2
   d. 2,4,1,3
   e. 2,4,1,3

<u>Questions 10 to 13 are 'true or false' type</u>
<u>Please state TRUE or FALSE in the space provided</u>

10. Low calorie diet with vegetables and fruits is advised in the management of Diabetes Mellitus _____
11. Exercise- increases insulin resistance. It also increases the blood glucose levels as muscle cell cannot utilize glucose without the help of insulin during exercise _____
12. Oral hypoglycemic agents decrease insulin secretion by inhibiting pancreas ______
13. Exogenous Insulin is very important in the treatment of Type I Diabetes Mellitus ______

<u>Questions 14 to 18 are 'fill in the blanks' type</u>
<u>Please CHOOSE the appropriate answer from the options given below and fill in the blanks</u>
<u>Options can also be used more than once</u>

14. Insulin Promotes _________
15. Insulin Inhibits _________
16. Insulin _____ formation of ketone bodies in the body
17. Insulin helps in the _________ of amino acids into the cells
18. Glucagon Promotes _________

lipogenesis	favours	entry
lipolysis	Inhibits	exit

19. Please select the appropriate options about insulin secretion from the following statements
Write your options in the box given below

a. GLUT 2 receptors in the beta cell allows glucose to enter the cell
b. GLUT 4 receptors in the beta cell allows glucose to enter the cell
c. Closure of ATP sensitive potassium channels
d. Closure of Calcium channels
e. Closure of voltage gated sodium channels
f. Opening of ATP sensitive potassium channels
g. Opening of Calcium channels
h. Opening of voltage gated sodium channels

20. Please select the appropriate options about functions of glucagon from the following
Write your options in the box given below

a. Lipolysis
b. Lipogenesis
c. Glycogenolysis
d. Gluconeogenesis
e. Glycogenesis
f. Protein synthesis
g. Protein lysis
h. Growth promoting effect
i. Growth inhibiting effect

21. **A 35 year old male came to a General Physician with complaints of increased thirst, frequent urination, increased hunger and weight loss for the past 6 months. After blood investigations, he was diagnosed to be diabetic and advised with life style changes and started on oral hypoglycaemic drugs.**

I. What could have been his fasting and 2-h plasma glucose levels (WHO criteria) to diagnose him to be a diabetic?

a. Fasting plasma glucose > 126mg/dl and 2-h plasma glucose>200mg/dl
b. Fasting plasma glucose > 120mg/dl and 2-h plasma glucose>156mg/dl
c. Fasting plasma glucose > 116mg/dl and 2-h plasma glucose>136 mg/dl
d. Fasting plasma glucose > 106mg/dl and 2-h plasma glucose>120 mg/dl

II. What could be the type of Diabetes he is suffering from?

a. Type 1
b. Type II

22. A recently diagnosed 40 year old diabetic was asked to exercise for 30 mins/day for at least 5 days a week by his general Physician.
Will it have an impact on his Blood glucose levels?

a. yes, during exercise, muscle cells utilize glucose without the need of insulin.
b. yes, during exercise, increased insulin secretion occurs from pancreas
c. No, during exercise, muscle cells release glucose.
d. No, during exercise, pancreas release glucagon.

23. A 40 year old male with severe uncontrolled diabetes complaints of weight loss, loss of energy in spite of eating large amount of food (polyphagia).
What could be the patho-physiology behind his symptoms?

a. Due to shift of carbohydrate metabolism to Fat metabolism due to lack of insulin
b. Due to shift of carbohydrate metabolism to Fat metabolism due to lack of glucagon
c. Due to shift of carbohydrate metabolism to Fat metabolism due to increased secretion of insulin
d. Due to shift of carbohydrate metabolism to Fat metabolism due to increased secretion of glucagon

24. A 65 year old woman with uncontrolled diabetes came with the complaints of severe thirst and large amounts of frequent urination to her general practitioner.
What could be the cause of her complaints?

a. Osmotic diuresis due to excess renal tubular glucose leading to polyuria and polydipsia
b. Osmotic diuresis due to excess ketoacid loss in urine leading to polyuria and polydipsia
c. Facilitated diffusion due to excess renal glucose leading to polyuria and polydipsia
d. Facilitated diffusion due to excess ketoacids leading to polyuria and polydipsia

25. A 16 year old boy was admitted to Emergency Room with signs of Diabetic ketoacidosis. His blood investigations showed high levels of blood glucose and ketoacids. What could be the level of C peptide in his blood sample?

a. Normal
b. Elevated
c. Negligible

e-module assessment questions

Topic: 'Juxta Glomerular Apparatus'

Name:__________________________________ Roll No:________

Multiple choice questions
Questions 1- 9have single BEST response
Please encircle the correct answer

1. The stretch receptors of Juxta glomerular apparatus
a. Monitor NaCl load at the distal tubule
b. Monitor arterial blood pressure
c. Monitor renal perfusion pressure
d. Monitor Extracellular fluid volume

2. The chemoreceptors which sense decreased NaCl concentration at the distal tubule
a. Juxta glomerular cells
b. macula densa cells
c. mesangial cells
d. principal cells

3. All of the following statements are true about Tubulo glomerular feedback *except*
a. Ensures constant sodium chloride delivery at the distal tubules
b. Autoregulates Glomerular Flitration rate
c. Autoregulates renal blood flow
d. ensures constant arterial blood pressure

4. Angiotensin II subserves the following functions *except*
a. Stimulates aldosterone secretion by adrenal cortex
b. Increases blood pressure by arteriolar vasoconstriction
c. Enhances NaCl reabsorption by proximal tubules
d. Inhibits ADH secretion and thirst

5. The following condition which increase the renin secretion
a. ↓ in Renal perfusion pressure
b. ↑ in Renal perfusion pressure
c. ↑ NaCl load at macula densa
d. ↓ Prostagladins

6. A 60 year old recently diagnosed male hypertensive was prescribed with Angiotensin II blockers to control the Blood Pressure, after 2 weeks of treatment his blood pressure has come under control . The Pharmacophysiology underlying the reduction in blood pressure by Angiotensin II blockers is
a. Reduction in Nacl reabsorption due to decreased levels of Angiotensin II
b. Increase in Nacl reabsorption due to decreased levels of Angiotensin II
c. Increase in Nacl reabsorption due to decreased levels of aldosterone
d. Reduction in Nacl reabsorption due to Increased levels of aldosterone

7. Juxtaglomerular cells are located in

a. Distal tubule which comes in contact with afferent and efferent arterioles
b. Proximal tubule which comes in contact with afferent and efferent arterioles
c. Afferent arterioles which comes in contact with proximal tubule
d. Afferent arterioles which comes in contact with distal tubule

8. Most of the conversion of Angiotensin I to Angiotensin II occurs in the endothelial cells of

a. lung　　b. liver　　c. kidney　　d. Heart.

9. Renin converts

a. angiotensinogen to angiotensin I
b. angiotensin I to angiotensin II
c. prostaglandins to angiotensinogen
d. angiotensinogen to angiotensin II

Q10 and Q 11: True or False type
Please state true or false in the space provided

10. Decreased sodium chloride concentration at Macula densa causes increase in renin release and dilatation of afferent arteriole ________

11. Tubulo – Glomerular feedback mechanism maintains GFR effectively even with large fluctuation in arterial blood pressure between 95 mmHg and 160 mmHg _________

Q12 to Q 18: Match the following type
Please write the appropriate number against the correct option

12. Macular Densa	i) converts angiotensinogen to angiotensin I
13. Juxta Glomerular cells	ii) Macular Densa
14. Lacis Cells	iii) Liver
15. Angiotensin Converting Enzyme	iv) Sodium Chloride in DCT
16. Angiotensinogen	v) Enalapril
17. Renin	vi) converts angiotensin I to angiotensin II
18. ACE inhibitor	vii) In the triangle between afferent and efferent arterioles
	viii) secrete renin
