# Supplementary material for: E-Learning Modules Based on Bloom Taxonomy and the Miller Pyramid for First-Year Indian Medical Students: Randomized Controlled Study in Medical Education
Source: JMIR Hum Factors. 2026 Apr 7;13:e84339. doi: 10.2196/84339 (PMC13055945; doi:10.2196/84339)
Supplement: Multimedia Appendix 8 [file humanfactors-v13-e84339-s008.pdf]

Validation Exercise

E- Module – High Altitude Physiology

We request you to validate this E- module developed by Dept. of Physiology, SRMC & RI as part of their Ph D program. Please give your feedback and valuable suggestions on the following parameters. Your responses will be maintained confidential. We thank you for your time and expertise.

Name of the educational expert: **Designation**:

Institutional affiliation:

Any other relevant information:

**Parameters**	**Agree**	**Disagree**	**Can be improved**
The objectives of the session are defined			
The contents match the learning objectives			
The E- module presents relevant information on the proposed topic			
The clarity of the E- module is good			
The overall organization of the E module follows appropriate sequence			
The images and animations have followed standardized illustrations			
The images and animations added to the E-module are relevant and appropriate for understanding the content			
Size and font of the text is appropriate			
Quality of the audio is good			
E module provides Proper feedback to the students on their performance (interactive assessment)			
Navigation of the keys are easy and user friendly			
Overall this e-module facilitate the learner to understand the proposed topic			

1. Overall **strength** of this E - Module:

2. **Limitations** of this E- module:

3. What are your **suggestions** for the improvement of the E - module?

Space for other comments:

Signature of the expert:

Validation Exercise

E- Module – Diabetes Mellitus

We request you to validate this E- module developed by Dept. of Physiology, SRMC & RI as part of their Ph D program. Please give your feedback and valuable suggestions on the following parameters. Your responses will be maintained confidential. We thank you for your time and expertise.

Name of the educational expert: **Designation**:

Institutional affiliation:

Any other relevant information:

**Parameters**	**Agree**	**Disagree**	**Can be improved**
The objectives of the session are defined			
The contents match the learning objectives			
The E- module presents relevant information on the proposed topic			
The clarity of the E- module is good			
The overall organization of the E module follows appropriate sequence			
The images and animations have followed standardized illustrations			
The images and animations added to the E-module are relevant and appropriate for understanding the content			
Size and font of the text is appropriate			
Quality of the audio is good			
E module provides Proper feedback to the students on their performance (interactive assessment)			
Navigation of the keys are easy and user friendly			
Overall this e-module facilitate the learner to understand the proposed topic			

1. Overall **strength** of this E - Module:

2. **Limitations** of this E- module:

3. What are your **suggestions** for the improvement of the E - module?

Space for other comments:

Signature of the expert:

Validation Exercise

E- Module – Juxta Glomerular Apparatus

We request you to validate this E- module developed by Dept. of Physiology, SRMC & RI as part of their Ph D program. Please give your feedback and valuable suggestions on the following parameters. Your responses will be maintained confidential. We thank you for your time and expertise.

Name of the educational expert: **Designation**:

Institutional affiliation:

Any other relevant information:

Parameters	Agree	Disagree	Can be improved
The objectives of the session are defined			
The contents match the learning objectives			
The E- module presents relevant information on the proposed topic			
The clarity of the E- module is good			
The overall organization of the E module follows appropriate sequence			
The images and animations have followed standardized illustrations			
The images and animations added to the E-module are relevant and appropriate for understanding the content			
Size and font of the text is appropriate			
Quality of the audio is good			
E module provides Proper feedback to the students on their performance (interactive assessment)			
Navigation of the keys are easy and user friendly			
Overall this e-module facilitate the learner to understand the proposed topic			

1. Overall **strength** of this E - Module:

2. **Limitations** of this E- module:

3. What are your **suggestions** for the improvement of the E - module?

Space for other comments:

Signature of the expert:
