# Supplementary material for: E-Learning Modules Based on Bloom Taxonomy and the Miller Pyramid for First-Year Indian Medical Students: Randomized Controlled Study in Medical Education
Source: JMIR Hum Factors. 2026 Apr 7;13:e84339. doi: 10.2196/84339 (PMC13055945; doi:10.2196/84339)
Supplement: Multimedia Appendix 10 [file humanfactors-v13-e84339-s010.pdf]

Feedback Form

E - module on ‘ Diabetes Mellitus’

Please give your feedback for the following parameters

Your responses will be maintained confidential

Name of the Session	Strongly agree	Agree	No comments	Disagree	Strongly disagree
1. The objectives of the session were clearly stated in the e - module					
2. The objectives of the session were met					
3. The format of the e – module suited my learning style					
4. The quality of e - module was pleasing					
5. The quality of animation was engaging					
6. The quality of audio was good					
7. The size and color of the font were appealing					
8. The content was tailored to meet the objectives					
9. The e – module was clear and informative					
10. The images added to the e - module were appropriate					
11. Adequate time was provided for all the components					
12. Venue and ambience were appropriate					
13. The e-module presentation provoked my interest					
14. I would like to have more e – modules for other sessions too					

1. What one thing you **liked the most** about the e-module presentation?

2. What one thing you **did not like** about the e -module presentation?

3. What could have been done **better** in the e - module presentation?

4. What are your **suggestions** for the improvement of the e –module presentation?

Space for other comments:

Feedback Form

JUXTAGLOMERULAR APPARATUS

Please give your feedback for the following parameters

Your responses will be maintained confidential

**Name of the Session**	**Strongly agree**	**Agree**	**No comments**	**Disagree**	**Strongly disagree**
The objectives of the session defined and stated					
The contents matched the learning objectives					
The E- module presentation was clear and structured					
I completed the assigned E- module prior to coming to class					
Learning key foundational content prior to coming to class greatly enhanced my learning in class.					
Interactive, applied in-class activities greatly enhanced my learning					
In-class discussions of course concepts with my peers greatly enhanced my learning.					
The images and animations added to the E-module were appropriate					
The format of the Flipped class E-module suited my learning style					
The quality of E-module was pleasing					
The E-module presentation provoked my interest					
I would like to have more E-module presentations for other sessions too					

1. What one thing you **liked the most** about the Flipped class- E module presentation?

2. What one thing you **did not like** about the Flipped class- E module presentation?

3. What could have been done **better** in the Flipped class- E module presentation?

4. What are your **suggestions** for the improvement of the Flipped class- E module presentation?

Space for other comments:
