# Supplementary material for: E-Learning Modules Based on Bloom Taxonomy and the Miller Pyramid for First-Year Indian Medical Students: Randomized Controlled Study in Medical Education
Source: JMIR Hum Factors. 2026 Apr 7;13:e84339. doi: 10.2196/84339 (PMC13055945; doi:10.2196/84339)
Supplement: Multimedia Appendix 11 [file humanfactors-v13-e84339-s011.pdf]

Supplementary file 9

Results of evaluation of Kirkpatrick's learning evaluation Model: Level 1 - Reaction

Table 1: Overall feedback about the e-modules

Items	N (320)	Strongly agree	Agree	Neutral	Disagree	Strongly disagree
The objectives of the session were clearly stated in the e-module	n	150	155	12	2	1
	%	47	48	4	1	0
The objectives of the session were met	n	134	167	17	2	0
	%	42	52	5	1	0
The format of the e-module suited my learning style	n	102	162	44	11	2
	%	32	51	14	3	1
The quality of the e-module was pleasing	n	80	140	54	40	6
	%	25	44	17	13	2
The quality of graphics and animation was engaging	n	91	129	88	12	0
	%	28	40	28	4	0
The quality of the audio was good	n	100	115	74	20	10
	%	31	36	23	6	3
The self-assessment was useful	n	95	169	39	17	0
	%	30	53	12	5	0
The content was tailored to meet the objectives	n	108	175	33	3	1
	%	34	55	10	1	0
The e-module was clear and informative	n	126	138	42	10	4
	%	39	43	13	3	1

.

Supplementary file 9

Results of evaluation of Kirkpatrick's learning evaluation Model: Level 1 - Reaction

Table 2: Feedback from the students regarding 'what they 'liked most' about the e-modules'

Themes emerged	Actual responses
Presentation<br>Animation<br>Audiovisual presentation<br>Attractive and catchy<br>Organization of information	'Content and images were amazing … thank you for making it'<br>'E module presentation… makes it interesting to us'<br>'We listen more'<br>'Its more colorful'<br>'I like the audio and pictures'<br>'The animations promote my understanding'
Interactive nature<br>Interesting<br>Easy to understand<br>Interactive assessment	'Could understand more clearly'<br>'It was interesting'<br>'Animations, questions and answer sessions at the end were very useful'

Table 3: Feedback from the students regarding 'what they 'did not like' about the e-modules'

Themes emerged	Actual responses
Quality of e-modules	'The font was too small'<br>'Technical problems'<br>'Audio wasn't that good otherwise it was perfectly a good session'<br>'e- module is not working for some people like me but I like the concept of e-module'
Length of the sessions	'Too long... Please make it shorter'<br>'Takes a lot of time'<br>'Too time consuming for one topic'<br>'Bit fast'
Interaction	'Could have been more interactive'<br>'I would have loved if more animations were there'<br>'No clarification by teachers/professors at home'<br>'We can't ask doubts with teachers or friends at home'<br>'Time for answering the question is less'

Supplementary file 9

Results of evaluation of Kirkpatrick’s learning evaluation Model: Level 1 - Reaction

Table 4: Feedback from the students regarding ‘suggestions for improvement of e-modules’

Themes emerged	Actual responses
Access without log in<br>Bigger font size<br>Shorter sessions<br>More animation<br>Increasing interactive sessions<br>Develop mobile application for e learning<br>Quality of e-modules	‘Sometimes logging in becomes difficult.. Can we access without logging in?’<br>‘I want mobile app for SRMC e-learning…please make it’<br>‘Fewer content and better colors‘<br>‘All are fine, it is my honest answer’<br>‘I think everything was good’<br>‘Play more video and audio’<br>‘It can be extended throughout this year..I like it’<br>‘The presentation with bigger font size’<br>‘It is very nice, some interesting videos can be placed for improvement’
