# Supplementary material for: E-Learning Modules Based on Bloom Taxonomy and the Miller Pyramid for First-Year Indian Medical Students: Randomized Controlled Study in Medical Education
Source: JMIR Hum Factors. 2026 Apr 7;13:e84339. doi: 10.2196/84339 (PMC13055945; doi:10.2196/84339)
Supplement: Multimedia Appendix 9 [file humanfactors-v13-e84339-s009.pdf]

Gmail

Archana Prabu Kumar <archanaprabukumar@gmail.com>

Automatic reply: Request for permission to use self directed learning readiness scale

Murray Fisher <murray.fisher@sydney.edu.au> Fri, Sep 23, 2016 at 8:06 AM
To: Archana prabu kumar <archanaprabukumar@gmail.com>

Dear Archana

Instrument attached for your use in the stated research.

[Quoted text hidden]

SELF-D~2.DOC
69K

Gmail

Archana Prabu Kumar <archanaprabukumar@gmail.com>

VARK Copyright Permission Request

2 messages

Neil Donald Fleming <neil.fleming@vark-learn.com> Wed, May 6, 2015 at 4:34 AM
To: "Dr.Archana P Kumar" <archanaprabukumar@gmail.com>

Dear Dr Archana P Kumar

BREAKING NEWS: We are planning some webinars on VARK for our researchers, education users and business trainers. If you would like us to keep you informed about these just send us an email. You may be able to clarify your research goals and avoid some pitfalls. The first one, with restricted numbers (<30), is likely to be free.

Thank you for seeking permission to use VARK™ for your research. We welcome VARK users and we appreciate the honesty of people to act in a professional way when using our copyright and trademarked materials.

Restrictions: You may not place VARK copyright materials online or on an electronic survey instrument, or any website, intranet or password protected site. This applies to using VARK for research, and all publications, free resources and for all resources made for sale, or for which fees are charged.

If you are a student or teacher in a high school, college or university you are welcome to use the VARK™ questionnaire for research by linking to our website You may also use paper copies. We ask that you provide this acknowledgement:
© Copyright Version 7.8 (2014) held by VARK Learn Limited, Christchurch, New Zealand.

Also free Research Package has been attached for you.

Gathering your Data: We can assist. If you are using paper copies of the VARK questionnaires for your research we can analyze your data into the VARK Categories for free. We have two scoring algorithms and one is designed specifically for research using standard deviations. The information about each system is at these web addresses.

http://www.vark-learn.com/english/page.asp?p=whatsnew
www.vark-learn.com/english/page.asp?p=advice

If, using paper copies of VARK is not appropriate, and, as you are not permitted to place VARK copyright resources on any online or electronic site, we can gather your data for you. Our system does not need any installation on your IT system. You get to manage the site and to download your results. The VARK Subscription Service is demonstrated on our website and the cost for six months for a research project is approximately $US95.

Book Downloads: You may find the VARK books helpful. They are all available as immediate and inexpensive downloads. They are sent immediately after payment, so don't shut down your computer until the book arrives as a PDF on your browser.

Business users should visit our VARK business site at: http://business.vark-learn.com

Best wishes for your research.

Neil

Neil D Fleming
Designer of the VARK Questionnaire
Director: VARK LEARN Limited
50 Idris Road, Christchurch 8052
New Zealand
www.vark-learn.com
phone: (64) 3 3517798

2 attachments

VARK Scoring compared.pdf
114K

A VARK RESEARCH PACKAGE.pdf
160K

Archana prabu kumar <archanaprabukumar@gmail.com> Wed, May 6, 2015 at 10:38 AM
To: Neil Donald Fleming <neil.fleming@vark-learn.com>

Dear Fleming,

Thank you very much for replying to my mail.
I shall follow all the guidelines in my educational research to the best of my knowledge.

Thanking you once again

with regards
Archana P Kumar
[Quoted text hidden]
--
Dr.Archana P Kumar
PSG FRI (2014 - New Batch)
Assistant Professor (Selection Grade)
Department of Physiology
Sri Ramachandra Medical College & Research Institute
Chennai - 600116
Mobile: +91 9962006869
